# Molecular diagnoses in the congenital malformations caused by ciliopathies cohort of the 100,000 Genomes Project

**DOI:** 10.1136/jmedgenet-2021-108065

**Published:** 2021-10-29

**Authors:** Sunayna Best, Jenny Lord, Matthew Roche, Christopher M Watson, James A Poulter, Roel P J Bevers, Alex Stuckey, Katarzyna Szymanska, Jamie M Ellingford, Jenny Carmichael, Helen Brittain, Carmel Toomes, Chris Inglehearn, Colin A Johnson, Gabrielle Wheway, Genomics England Research Consortium, John C Ambrose

**Affiliations:** 1 Division of Molecular Medicine, University of Leeds Leeds Institute of Medical Research at St James's, Leeds, West Yorkshire, UK; 2 Department of Clinical Genetics, Leeds Teaching Hospitals NHS Trust, Leeds, UK; 3 Department of Human Development and Health, University of Southampton Faculty of Medicine, Southampton, UK; 4 University Hospital Southampton NHS Foundation Trust, Southampton, UK; 5 Mid Yorkshire Hospitals NHS Trust, Wakefield, UK; 6 Department of Yorkshire Regional Genetics Service, Leeds Teaching Hospitals NHS Trust, Leeds, West Yorkshire, UK; 7 School of Medicine, University of Leeds, Leeds, UK; 8 Genomics England, Queen Mary University of London, London, UK; 9 Division of Evolution and Genomic Sciences, Faculty of Biology, Medicine and Health, University of Manchester, Manchester, UK; 10 Manchester Centre for Genomic Medicine, Manchester, UK; 11 East Anglian Medical Genetics Service, Addenbrooke’s Hospital, Cambridge, UK; 12 Southampton University Hospitals NHS Trust, Southampton, UK

**Keywords:** genetics, medical, genomics, congenital, hereditary, and neonatal diseases and abnormalities, diagnosis

## Abstract

**Background:**

Primary ciliopathies represent a group of inherited disorders due to defects in the primary cilium, the ‘cell’s antenna’. The 100,000 Genomes Project was launched in 2012 by Genomics England (GEL), recruiting National Health Service (NHS) patients with eligible rare diseases and cancer. Sequence data were linked to Human Phenotype Ontology (HPO) terms entered by recruiting clinicians.

**Methods:**

Eighty-three prescreened probands were recruited to the 100,000 Genomes Project suspected to have congenital malformations caused by ciliopathies in the following disease categories: Bardet-Biedl syndrome (n=45), Joubert syndrome (n=14) and ‘Rare Multisystem Ciliopathy Disorders’ (n=24). We implemented a bespoke variant filtering and analysis strategy to improve molecular diagnostic rates for these participants.

**Results:**

We determined a research molecular diagnosis for n=43/83 (51.8%) probands. This is 19.3% higher than previously reported by GEL (n=27/83 (32.5%)). A high proportion of diagnoses are due to variants in non-ciliopathy disease genes (n=19/43, 44.2%) which may reflect difficulties in clinical recognition of ciliopathies. n=11/83 probands (13.3%) had at least one causative variant outside the tiers 1 and 2 variant prioritisation categories (GEL’s automated triaging procedure), which would not be reviewed in standard 100,000 Genomes Project diagnostic strategies. These include four structural variants and three predicted to cause non-canonical splicing defects. Two unrelated participants have biallelic likely pathogenic variants in *LRRC45*, a putative novel ciliopathy disease gene.

**Conclusion:**

These data illustrate the power of linking large-scale genome sequence to phenotype information. They demonstrate the value of research collaborations in order to maximise interpretation of genomic data.

## Introduction

Ciliopathies represent a group of inherited genetic disorders that arise as a result of defects in the primary cilium, the ‘cell’s antenna’,^1^ or motile cilia, organelles responsible for the movement of fluid over the surface of cells.^2^ They encompass a range of severe developmental and degenerative diseases that are individually rare but collectively common, affecting an estimated 15.8 million people worldwide including an estimated 133 000 people in the UK. Cilia have also been implicated in conditions such as diabetes, cancer, congenital heart disease and osteoarthritis.^3–5^ As cilia have a near-ubiquitous anatomical distribution, genetic defects affecting the structure or function of cilia cause a range of conditions that can affect multiple organs. Ciliopathies are typically classified into: retinal ciliopathies that exclusively or predominantly affect the eye^6^; renal ciliopathies, which include autosomal dominant polycystic kidney disease affecting around 1:500 people^7^; skeletal ciliopathies that cause a diverse range of skeletal dysplasias and cranio-facial dysmorphology^8^; metabolic or ‘obesity’ ciliopathies^9^; neurodevelopmental ciliopathies^10^; and the respiratory motile ciliopathies.^11^

It is estimated that around 1000 genes contribute to ciliogenesis and cilium function,^12–15^ and ciliopathies are highly genetically heterogeneous.^16 17^ Approximately one-third of the around 270 genes implicated in inherited retinal dystrophies are cilia genes,^18^ whereas roughly 20 genes have been associated with renal ciliopathies (PKD OMIM phenotypic series PS173900; nephronophthisis OMIM PS256100). The short-rib polydactyly syndromes, which encompass most of the skeletal ciliopathies, have 22 known genetic causes (OMIM PS208500). There are 24 known genetic causes of the metabolic/obesity ciliopathy Bardet-Biedl syndrome (BBS) (OMIM PS209900). In this same series, Alström syndrome is unusual, because it is a single gene ciliopathy (caused by pathogenic variants in *ALMS1*). There is extensive genetic overlap between neurodevelopmental ciliopathies Joubert syndrome (JBTS) and Meckel-Gruber syndrome (MKS), with 37 known JBTS genes (OMIM PS213300) and 13 MKS genes (OMIM PS249000), many of which also cause JBTS. Several MKS and JBTS disease genes also overlap with the nine genes known to cause complex multiorgan ciliopathy orofacial digital syndrome (OFD) (OMIM PS311200). OFD is considered by some to be a skeletal ciliopathy, involving malformations of the face, mouth and digits, while OFD type 1, which specifically includes polycystic kidney disease, may be considered a renal ciliopathy. In total, at least 220 different genes have been shown to cause a single (or multiple) ciliopathy when mutated.

The number of identified ciliopathy disease genes has advanced rapidly since the early to mid-2010s following the ubiquitous implementation of next-generation sequencing (NGS) technologies. Using targeted gene panel, or whole exome sequencing (WES) approaches, genetic diagnosis rates for syndromic primary (non-motile) ciliopathies are typically 40%–70% and for motile (respiratory ciliopathies) are approximately 70% (studies summarised in online supplemental table 1). A recent large whole genome sequencing (WGS) study in 125 families with ciliopathies achieved an 87% diagnosis rate,^16^ and a further increase was achieved following the inclusion of structural variant (SV) analysis and RNA sequencing in carefully phenotyped cohorts.^19^

10.1136/jmedgenet-2021-108065.supp1 Supplementary data

The 100,000 Genomes project is a hybrid clinical/research initiative, launched in 2012 and overseen by Genomics England Ltd (GEL), a company set up and wholly owned by the UK Government Department of Health and Social Care.^20^ The project aimed to sequence 100 000 genomes from 70 000 individuals with rare diseases and cancer. Rare disease patients’ genomes were sequenced alongside their family members in a trio testing approach. Cancer patients’ germline and somatic genomes were sequenced from matched tumour and normal tissue. Genome sequence data were linked to clinical data from longitudinal patient records and Human Phenotype Ontology (HPO) terms entered by recruiting clinicians. Participants consented to receive a diagnosis for the specific condition they were recruited to the project for and to allow access to their fully anonymised genome sequence data and phenotype information for approved academic and commercial researchers. Recruitment to 190 different rare disease domains took place between 2016 and 2018 across 85 NHS Trusts, coordinated by 13 Genomic Medicine Centres (GMCs). In the data release used in this study (Main Programme Release 11 (17 December 2020)), data were available for 88 918 individuals: 71 682 in the rare diseases arm of the 100,000 Genomes Project and 17 236 in the cancer arm. In the rare diseases arm, 33 329 participants were entered as probands and 38 352 as relatives.

GEL also developed PanelApp (available from https://panelapp.genomicsengland.co.uk), a crowdsourcing tool for sharing and evaluation of gene panels by the scientific community.^21^ Virtual gene panels were applied to WGS data to facilitate focused analysis, returning variants in selected genes on curated lists with convincing evidence of an association with the disease(s) of interest. Not only does this shorten the list of variants to analyse, but it also reduces the risk of unwanted incidental findings.

As part of the effort to integrate NGS into standard of care (SOC) testing in the UK’s National Health Service (NHS), ciliopathy patients who had previously undergone existing SOC testing (typically gene panel testing) were recruited to the 100,000 Genomes Project to undergo WGS.^22^ Patients recruited under congenital malformations caused by ciliopathies (CMC) categories (subdivided into BBS, JBTS and rare multisystem ciliopathy disorders (RMCD) or respiratory ciliopathies) accounted for just under 1% of the total rare disease cohort. There were no dedicated recruitment categories for retinal ciliopathies, renal ciliopathies or skeletal ciliopathies, and these were recruited under subcategories of ophthalmological disorders, renal and urinary tract disorders or other categories, and so there are likely to be many further ciliopathy participants in the rare disease cohort. In this study, we aimed to optimise strategies to improve molecular diagnostic rates for probands recruited to the CMC category within the 100,000 Genomes Project.

## Materials and methods

### Participant selection and phenotypic classification

Participants recruited under CMC categories were extracted from the GEL Main Programme Release 11 (17 December 2020) using the user interface ‘LabKey’ within the GEL secure research environment. All data analysis was conducted within the GEL Research Environment. We exported anonymised data for publication through the Airlock system, after review by the GEL Airlock Review Committee. HPO terms recorded for each participant by their recruiting clinicians were assessed within the research environment prior to genetic analysis to determine the most likely clinical diagnosis for each proband based on phenotypic features alone. For selected cases, further clinical information was obtained through the ‘Participant Explorer’ interface.

### Variant filtering and analysis

The GEL data processing pipeline, which includes an automated variant triaging algorithm to classify variants into a series of ‘Tiered’ categories (as defined by the Genomics England Rare Disease Tiering Process), has been described previously.^22^ Variants were tiered against ‘green’ genes listed in PanelApp panels selected according to entered HPO terms. PanelApp provides a traffic light system for genes: ‘green’ genes are diagnostic grade, ‘amber’ genes are borderline and ‘red’ genes have a low level of evidence. In instances where tiered variants did not indicate the cause of disease, untiered single nucleotide variants (SNVs) including heterozygous variants were extracted from participant genomes using a custom Python script (‘find_variants_by_gene_and_consequence.py’; available at https://github.com/JLord86/Extract_variants). The script extracts variants in diagnostic grade ‘green’ genes from provided PanelApp panels and candidate genes with the variant effect predictor (VEP) annotations stop_gained, splice_acceptor, splice_donor, frameshift, missense and splice_region (if the variant was within either the terminal 1–3 bases of the exon or terminal 3–8 bases of the intron).

The script was first run using the RMCD Super Panel V.4.91 (available from https://panelapp.genomicsengland.co.uk/panels/728/) (green genes recorded in online supplemental table 2) and ciliopathy candidate genes from several sources. These include all ‘red’ and ‘amber’ genes from the PanelApp RMCD panel, genes of interest highlighted by local research teams and all genes on the curated SYSCILIA gold standard (SCGSv1) (online supplemental table 3). If a single potentially pathogenic heterozygous SNV in a recessive gene was identified through this strategy, manual inspection of the whole gene locus was undertaken using the Integrative Genomics Browser (IGV)^23^ to determine if a potential SV could be identified as the second biallelic variant. SVs were considered potentially causative if present in >30% of reads.

For those cases that remained unsolved, untiered SNVs were then extracted using further panels compatible with the participant’s phenotype. These included: the Retinal Disorders panel V.2.172 for those with retinal dystrophy only (available from https://panelapp.genomicsengland.co.uk/panels/307/), the Developmental Disorders Genotype-to-Phenotype database (DDG2P) panel V.2.21 for those with multisystemic developmental disorders (https://panelapp.genomicsengland.co.uk/panels/484/), the Laterality Disorders and Isomerism panel V.1.21 for those with a laterality defect (https://panelapp.genomicsengland.co.uk/panels/549/) and the Broad Renal Super panel V.2.346 for those with isolated renal anomalies (https://panelapp.genomicsengland.co.uk/panels/902/).

For all remaining unsolved participants, variants potentially affecting splicing (SpliceAI delta scores >0.5) in diagnostic grade ‘green’ genes) from the PanelApp RMCD panel were extracted with a further custom Python script (‘find_variants_by_gene_and_SpliceAI_score.py’; available at https://github.com/JLord86/Extract_variants).^24^ Finally, the find_variants_by_gene_and_SpliceAI_score.py Python script was run again using the DDG2P panel V.2.21 for all remaining unsolved participants.

### Bespoke research variant analysis pipeline

All data anlysis was conducted within the secure online Research Environment including interrogation of BAM, VCF, SV and HPO information files. The Ensembl VEP was used to obtain variant information for interpretation of variant pathogenicity.^25^ Information about associations between genes and disease phenotypes was obtained from the OMIM database (https://www.omim.org). The mode of inheritance was defined according to the literature and OMIM for each gene. Variant evidence was reviewed using ACMG/AMP guidelines for clinical variant interpretation,^26^ and each variant of interest was assigned a pathogenicity score according to current (Association for Clinical Genomic Science (ACGS) guidelines.^27^

**Figure 1:**
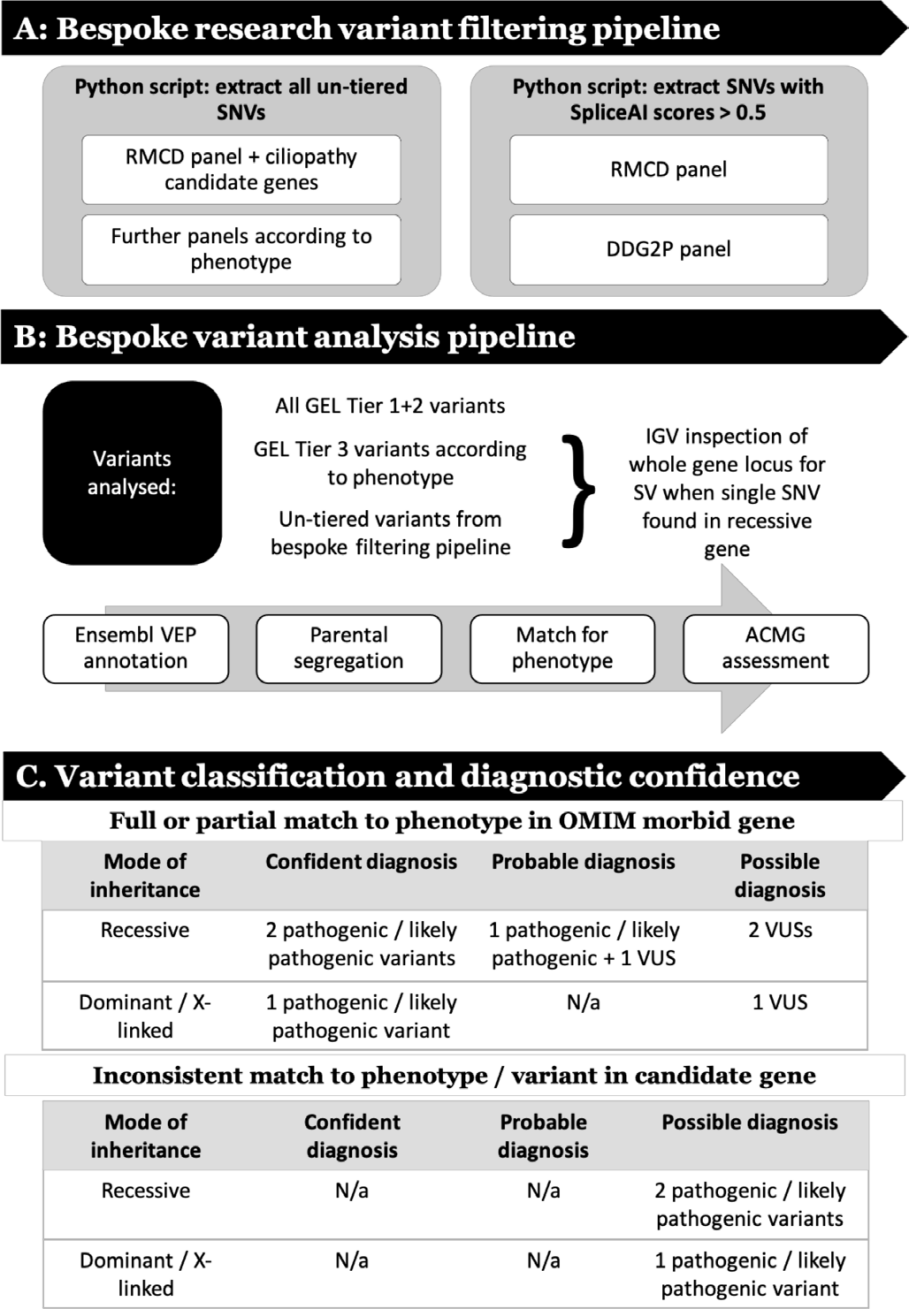
Research analysis workflow that (A) describes steps to filter genomic data, (B) analyse putative pathogenic variants and (C) classify variants then assign diagnostic confidence. ACMG, Association for Clinical Genomic Science; DDG2P, Development Disorder Genotype - Phenotype Database; GEL, Genomics England; IGV, Integrative Genomics Browser; RMCD, rare multisystem ciliopathy disorders; SNV, single nucleotide variant; SV, structural variant; VEP, variant effect predictor; VUS, variant of uncertain significance.

The research analysis workflow comprised steps to filter genomic data (figure 1A), assess putative pathogenic variants (figure 1B), then classify and assign diagnostic confidence (figure 1C).

### Variant classification and diagnostic confidence

To benchmark our ability to appropriately classify and interpret identified variants, first-pass analysis was blinded to previous results, and then verified against the GEL reported findings in the GMC exit questionnaires. These were completed by regional NHS GMCs for each analysed participant. Recruiting clinicians were contacted through the GEL secure airlock system for notification of a research molecular diagnoses, if they did not have a consistent completed GMC exit questionnaire. Additional clinical data were requested, where required, using the ‘contact the clinician’ form. All diagnoses identified through this blinded research strategy were termed ‘research molecular diagnoses’. The interpretation of these findings was subdivided into ‘confident’, ‘probable’ or ‘possible’ according to the ACMG classification for each variant, the inheritance pattern of the identified condition and the match to the proband’s phenotypic features (summarised in figure 1C).

## Results

### Congenital malformations caused by ciliopathies cohort

A total of 83 probands were identified in the CMC cohort. This was subdivided into 45 in the BBS category, 14 in the JBTS category and 24 in the RMCD category. Fifteen participants were recruited as singleton cases, and for 68 individuals at least one additional family member underwent WGS. Including probands and relatives, genomic data were available for 211 individuals.

### HPO term analysis

Analysis of HPO terms for the 83 probands shows that for 51 cases, phenotypes were consistent with their disease recruitment category. The remaining 32 probands lack recorded phenotypes suggestive of a syndromic ciliopathy (table 1). This suggests that participants were either frequently misdiagnosed as having ciliopathies or HPO terms were not entered accurately.

**Table 1:** Anonymised phenotypic and research molecular diagnosis data for the probands in the congenital malformations caused by ciliopathies cohort

Research number	Recruitment category	Most likely clinical diagnosis based on HPO terms	Does recruitment category match most likely clinical diagnosis?	GEL GMC exit report	Research molecular diagnosis	Gene	Is identified diagnosis a ciliopathy?	Diagnostic confidence
1	JBTS	JBTS	Yes	Sol	CHARGE Syn	*CHD7*	No	Conf
2	BBS	Non-cil MS cond	No	Sol	Alström Syn	*ALMS1*	Yes	Conf
3	BBS	BBS	Yes	Sol	BBS +RP	*ARL6 +IMPG2*	Yes	Conf
4	BBS	BBS	Yes	Sol	RP	*RPGR*	Yes	Conf
5	BBS	Non-cil MS cond	No	Sol	Retinal cil, possibly syndromic	*CEP290*	Yes	Conf
6	JBTS	JBTS	Yes	Sol	JBTS	*KIAA0586*	Yes	Conf
7	RMCD	OFD-like cil	Yes	Sol	OFD1, PKD +inherited cataract	*OFD1, PKD1, CRYBB1*	Yes (*OFD1*)	*OFD1* Conf, *PKD1 +CRYYB1* Poss
8	BBS	Isol RD	No	Sol	RP	*PRPF8*	No	Conf
9	RMCD	JBTS-like MS cil	Yes	Uns	Seckel Syn	*CEP152*	No	Poss
10	JBTS	JBTS	Yes	Sol	JBTS	*CEP290*	Yes	Conf
11	RMCD	Jeune-like cil	Yes	Unr	Feingold Syn	*MYCN*	No	Conf
12	JBTS	JBTS	Yes	Unr	JBTS	*ARMC9*	Yes	Conf
13	BBS	BBS	Yes	Unr	Tubulinopathy	*TUBA1A*	No	Poss
14	RMCD	Jeune-like cil	Yes	Unr	Jeune Syn	*WDR19*	Yes	Conf
15	BBS	Isol RD	No	Unr	RP	*RHO*	No	Conf
16	RMCD	Non-cil MS cond	No	VUS	STAG1 syndromic ID syn	*STAG1*	No	Prob
17	BBS	BBS	Yes	Sol	BBS	*BBS1*	Yes	Conf
18	BBS	BBS	Yes	Sol	Neurodevelopmental disorder	*RERE*	No	Conf
19	BBS	BBS	Yes	Sol	Alström Syn	*ALMS1*	Yes	Conf
20	BBS	Isol eye cond (not RD)	No	Sol	BBS	*BBS2*	Yes	Conf
21	JBTS	JBTS	Yes	Unr	Poretti-Boltshauser Syn+Arboleda Tham Syn	*LAMA1, KAT6A*	No	*LAMA1* Prob, *KAT6A* Poss
22	BBS	BBS	Yes	Sol	BBS	*MKKS*	Yes	Conf
23	JBTS	JBTS	Yes	Sol	JBTS	*CEP290*	Yes	Prob
24	BBS	Non-cil MS cond	No	Uns				Uns
25	BBS	BBS	Yes	Sol	Smith Magenis Syn	*RAI1*	No	Conf
26	BBS	BBS	Yes	Sol	Cone-rod dystrophy	*PROM1*	No	Conf
27	JBTS	Non-cil MS cond	No	Unr	Luscan-Lumish Syn	*SETD2*	No	Conf
28	BBS	Non-cil MS cond	No	Sol	Optic Atrophy	*OPA1*	No	Conf
29	BBS	Non-cil MS cond	No	Sol	Alström Syn	*ALMS1*	Yes	Conf
30	BBS	BBS	Yes	Sol	Chung-Jansen Syn	*PHIP*	No	Conf
31	BBS	Isol RD	No	Sol	Cone-rod dystrophy	*RAB28*	Yes	Conf
32	BBS	BBS	Yes	Sol	None: Unsolved	*ALMS1*	N/a	False+ve
33	RMCD	Non-cil MS cond	No	Uns				Uns
34	RMCD	Non-cil MS cond	No	Uns	Van Esch-O'Driscoll Syn	*POLA1*	No	Poss
35	JBTS	JBTS	Yes	Uns				Uns
36	JBTS	JBTS	Yes	Uns				Uns
37	RMCD	Non-cil MS cond	No	Uns				Uns
38	BBS	BBS	Yes	Uns				Uns
39	BBS	BBS	Yes	Uns				Uns
40	BBS	BBS	Yes	Uns				Uns
41	JBTS	JBTS	Yes	Uns	JBTS	*CSPP1*	Yes	Prob
42	JBTS	JBTS	Yes	Unr	JBTS	*PIBF1*	Yes	Prob
43	BBS	BBS	Yes	Uns				Uns
44	RMCD	Non-cil MS cond	No	Uns				Uns
45	BBS	Isol polydactyly	No	Uns				Uns
46	RMCD	MKS/JBTS-like MS cil	Yes	Uns				Uns
47	BBS	Non-cil MS cond	No	Unr				Uns
48	RMCD	BBS-like MS cil	Yes	Uns	Candidate cil	*LRRC45*	Candidate	Poss
49	RMCD	Non-cil MS cond	No	Unr				Uns
50	BBS	BBS	Yes	Unr				Uns
51	RMCD	DM	DM	Unr				Uns
52	RMCD	JBTS-like MS cil	Yes	Unr				Uns
53	RMCD	Isol GI disorder	No	Unr				Uns
54	RMCD	Non-cil MS cond	No	Uns				Uns
55	JBTS	JBTS	Yes	Uns				Uns
56	BBS	Isol eye cond (not RD)	No	VUS	BBS	*BBS9*	Yes	Poss
57	JBTS	JBTS	Yes	Uns				Uns
58	RMCD	JBTS-like MS cil	Yes	Uns				Uns
59	BBS	BBS	Yes	Uns				Uns
60	BBS	BBS	Yes	Uns				Uns
61	RMCD	Non-cil MS cond	No	Unr	WT1-related disorder	*WT1*	No	Conf
62	RMCD	Non-cil MS cond	No	Uns				Uns
63	RMCD	Non-cil MS cond	No	Uns				Uns
64	RMCD	JBTS-like MS cil	Yes	Uns				Uns
65	BBS	BBS	Yes	Uns				Uns
66	RMCD	BBS-like MS cil	Yes	Uns				Uns
67	BBS	Non-cil MS cond	No	VUS	Alström Syn	*ALMS1*	Yes	Poss
68	JBTS	JBTS	Yes	Uns				Uns
69	BBS	BBS	Yes	Sol	BBS	*BBS1*	Yes	Conf
70	BBS	Non-cil MS cond	No	Uns				Uns
71	RMCD	Non-cil MS cond	No	Unr	Shukla-Vernon Syn	*BCORL1*	No	Poss
72	BBS	BBS	Yes	Unr	Sifrim-Hitz-Weiss Syn	*CHD4*	No	Poss
73	RMCD	Isol GI disorder	No	Uns				Uns
74	BBS	Non-cil MS cond	No	Uns				Uns
75	BBS	DM	DM	Unr	BBS	*BBS4*	Yes	Poss
76	BBS	BBS	Yes	VUS	BBS	*BBS10*	Yes	Poss
77	BBS	BBS	Yes	Uns				Uns
78	BBS	BBS	Yes	Uns				Uns
79	BBS	BBS	Yes	Uns				Uns
80	BBS	BBS	Yes	Uns				Uns
81	BBS	BBS	Yes	Uns				Uns
82	BBS	Non-cil MS cond	No	Unr	Attenuated mucopolysaccharidosis 1	*IDUA*	No	Prob
83	BBS	BBS	Yes	Uns				Uns

Table includes the recruitment category, designated ‘most likely’ clinical diagnosis based on entered HPO terms alone, GEL GMC exit questionnaire reporting outcome, research molecular diagnosis (determined by genotype), responsible gene, whether the identified diagnosis is a ciliopathy and diagnostic confidence. Note: individual variant information, including data taken into consideration in forming ACMG classifications, can be found in online supplemental table 4.

BBS, Bardet-Biedl syndrome; Cil, ciliopathy; Cond, condition; Conf, confident; DM, data missing; GEL, Genomics England; GI, gastrointestinal; GMC, Genomic Medicine Centres; HPO, Human Phenotype Ontology; Isol, isolated; JBTS, Joubert syndrome; MKS, Meckel Gruber syndrome; MS, multisystemic; PKD, polycystic kidney disease; Poss, possible; Prob, probable; RD, retinal dystrophy; RMCD, rare multisystem ciliopathy disorders; RP, retinitis pigmentosa; Sol, solved; Syn, syndrome; Unr, unreported; Uns, unsolved.

### Tiered variants

Thirty-eight tier 1 variants were identified in 28 different genes among 29 different probands in the CMC cohort. Two hundred and sixteen tier 2 variants were identified in 142 different genes among 53 different probands. A total of 8777 tier 3 variants were identified in 5220 different genes among all 83 probands. No SVs had been tiered.

### GEL reported molecular diagnoses

**Figure 2:**
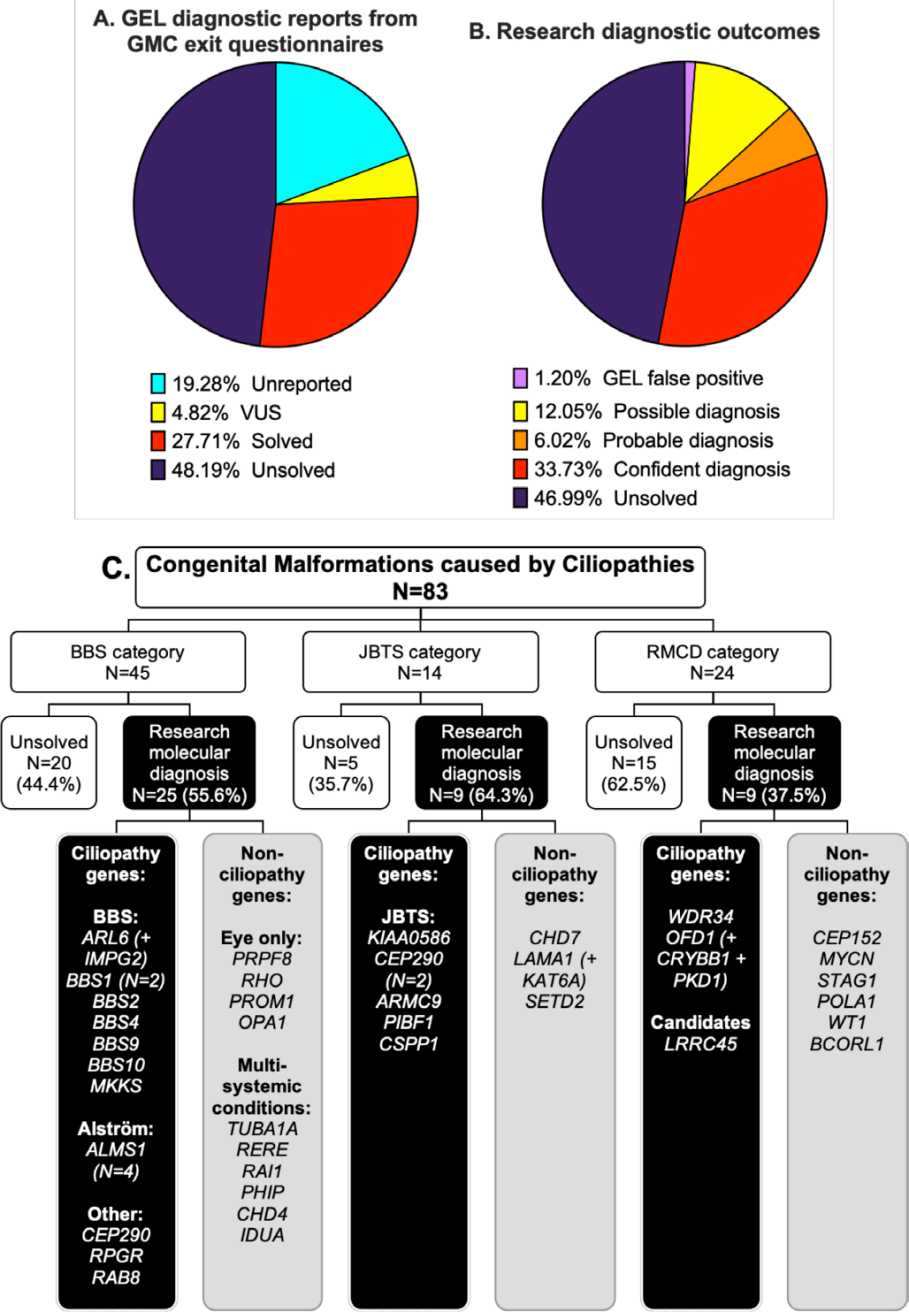
Comparison of diagnostic reporting outcomes between gel GMC exit reports (A) and research diagnostic outcomes (B) for the 83 probands in the CMC cohort. (C) Research molecular diagnoses according to recruitment category. Genes with identified potentially causative variants are grouped according to whether they are known to be associated with ciliopathies or not. A ‘+’ is used where participants had potentially causative variants in more than one gene contributing to their clinical features (additional gene(s) are included in brackets). Diagnostic confidence for each research molecular diagnosis is shown in table 1. Detailed variant information, including whether the gene variants(s) are thought to be a full or partial match to phenotype, is provided in online supplemental table 4. BBS, Bardet-Biedl syndrome; CMC, congenital malformations caused by ciliopathies; GEL, Genomics England; GMC, Genomic Medicine Centre; JBTS, Joubert syndrome; RMCD, rare multisystem ciliopathy disoder.

GMC exit questionnaires were completed for 67/83 (80.7%) patients by Release 11 (released 17 December 2020) (table 1). Twenty-three participants (27.7%) had GMC exit questionnaires reporting causative tier 1 or tier 2 variants, with one case partially solved and 22 fully solved. Four GMC exit questionnaires reported variants of uncertain significance (VUS) (figure 2A).

We identified that one of the cases previously reported as solved was a false positive. The GMC questionnaire reported compound heterozygous *ALMS1* variants in participant #32 including an untiered heterozygous exon 11 deletion. The deletion was not visible using the IGV or detectable in the patients VCF file; following correspondence with the GEL helpdesk. the variant was confirmed to be a false positive.

### Identification of research molecular diagnoses

Our bespoke variant-to-diagnosis pipeline shows that 43 of the 83 probands (51.8%) have a research molecular diagnosis that is compatible with their phenotypic features (table 1). Individual variant information, including data taken into consideration in performing ACMG classification, is recorded in online supplemental table 4. Twenty-eight of the 83 participants (33.7%) are classified as having a confident diagnosis, 5/83 (6%) a probable diagnosis and 10/83 (12%) only a possible diagnosis (figure 2B). Overall, 34/83 participants (41%) had a research molecular diagnosis that fully accounted for their entered phenotypic features and 9/83 (10.8%) that partially accounted for their entered features (online supplemental table 4). No phenotypic features were entered for proband #75, but the possible molecular diagnosis of BBS matches their BBS recruitment category. Diagnoses according to recruitment category are shown in figure 2C.

Seventeen of the 43 research molecular diagnoses (39.5%) can be considered novel findings. Fourteen diagnoses are new findings in probands with no completed GMC exit questionnaire (unreported) and three are in probands with negative GMC outcome questionnaires (reported as ‘unsolved’). Interestingly, a significant proportion of research molecular diagnoses have been made in non-ciliopathy genes. Only 23 of the 43 potentially solved participants (53.5%) have variants in genes known to be causative of ciliopathy syndromes. The remaining 19/43 potentially solved probands (44.2%) have variants identified in non-ciliopathy genes.

### Research molecular diagnoses made outside GEL tiers 1 and 2

**Figure 3:**
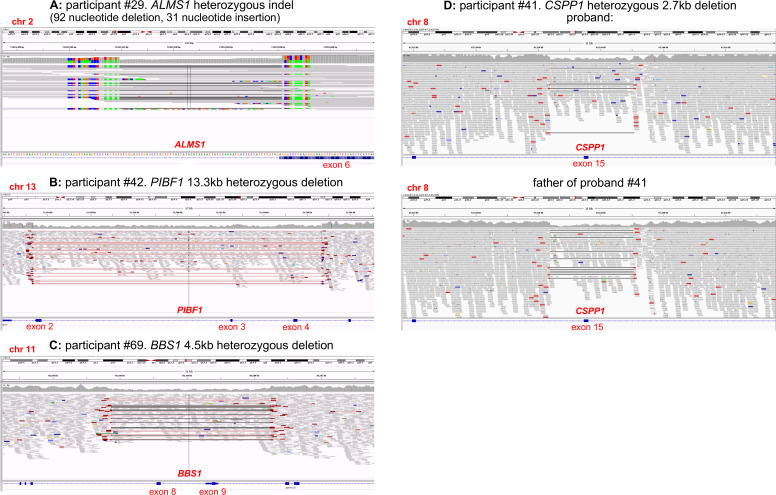
IGV captures of structural variants identified among participants of the congenital malformations caused by ciliopathies cohort. First, an untiered *ALMS1* SV identified in participant #29 was initially called a tier 2 *ALMS1* missense variant. Closer inspection on IGV determined that this was an indel (92 nucleotide deletion and 31 nucleotide insertion) leading to a splice acceptor change at the beginning of exon 6 (A). Our filtering pipeline identified a second untiered *ALMS1* frameshift variant, completing the molecular diagnosis of Alström syndrome. Three larger heterozygous deletions were identified through manual IGV inspection of whole gene loci when searching for second hits in probands with potentially causative SNVs. An untiered 13.3 kb deletion in *PIBF1* (also known as *CEP90*) (B) was identified in a proband with an untiered novel missense variant (proband #42). An untiered 4.5 kb deletion in *BBS1* (C) was found in a proband with an untiered, ClinVar pathogenic missense variant (proband #69). Finally, a 2.7 kb deletion in *CSPP1* (D) was found in a proband with a predicted splice donor loss (SpliceAI DS_DL 0.79) (proband #41). This *CSPP1* deletion was only seen in ~30% of reads in the proband but in ~50% of reads in their father. SNV, single nucleotide variant.

Thirty-two of the 83 probands (38.5%) have research molecular diagnoses made from tier 1 and 2 variants only. The remaining 11/83 probands (13.3%) with research molecular diagnoses have at least one variant outside of tiers 1 and 2 (variant information provided in online supplemental table 4). These diagnoses would have been missed by the standard 100,000 Genomes Project diagnostic pipeline, which routinely inspects only tier 1 and 2 variants. Five tier 3 variants and 12 untiered variants contribute to the diagnoses for these 11 participants. Three of the untiered variants are SVs (IGV captures shown in figure 3); the other nine are SNVs identified through our bespoke filtering pipeline. Interestingly, a variant annotated by GEL as a tier 2 *ALMS1* missense was discovered via IGV inspection to be an indel (92 nucleotide deletion and 31 nucleotide insertion) leading to a splice acceptor change (participant #29, shown in figure 3A).

SpliceAI analysis of variants filtered using our pipeline identified three untiered ciliopathy gene variants predicted to cause splice donor site losses. One is a homozygous synonymous variant in *ARL6* in proband #3, entered with suspected BBS (NM_001278293.3:c.534A>G, NP_001265222.1:p.Gln178=) (online supplemental table 4). The overall allele frequency (AF) on gnomAD is 0.000007960 with zero homozygotes.^28^ The 100,000 Genomes Project AF is 0.00049985 for participants called on GrCh37 (one heterozygote) and 0.0000571872 for participants called on GrCh38 (three heterozygotes and three homozygotes). On further analysis, the two further homozygous individuals were identified as affected siblings of proband #3. The heterozygous individuals are the parents of proband #3 plus one unrelated participant. This variant has previously been published in association with BBS and proven to cause aberrant splicing in vitro by minigene assay.^29^ The other two are at +3 and +5 positions in probands #75 (*BBS4* NM_033028.5:c.642+3A>T) and #41 (*CSPP1* NM_001382391.1:c.2968+5G>A). Clinical material was not available for testing to validate splicing effects at the molecular level. Therefore, both have been classified as VUSs.

### Putative novel disease genes

Participant #48, entered to the RMCD category and determined most likely to have BBS based on entered HPO terms, has two separate homozygous, protein-truncating variants in candidate ciliopathy genes. Proband #48 has a sibling who was separately entered to the 100,000 Genomes Project in the intellectual disability category, without additional features suggestive of a syndromic ciliopathy. Further phenotypic analysis using the Participant Explorer tool revealed that participant #48 also has clinical features suggestive of a motile ciliopathy. Specific clinical features cannot be provided to protect participant anonymity. There is a recorded history of parental consanguinity in this family.

The first variant of interest identified in participant #48 is a homozygous frameshift variant in *LRRC45* (GrCh38 chromosome 17: 82028260 C>CTG; NM_144999.4:c.1074_1075insTG, NP_659436.1:p.Leu359CysfsTer19). This was also found to be homozygous in the proband’s sibling from the intellectual disability category. Segregation analysis is consistent with autosomal recessive inheritance; both parents are confirmed heterozygotes. According to the Illumina Region of Homozygosity (ROH) caller, this *LRRC45* variant is in a 1 359 569 base pair ROH (GrCh38 chromosome 17: 81841582–83201151) containing 797 homozygous and zero heterozygous variants (ROH score 19.92) in the proband and an 1 364 960 base pair ROH (GrCh38 chromosome 17: 81841582–83206542) containing 728 homozygous and zero heterozygous variants (ROH score 18.2) in the sibling. The second variant of interest is a homozygous stop gain variant in *CFAP45 (CCDC19*) (GrCh38 chromosome 1: 159 887 996 G>A; NM_012337.3:c.433C>T, NP_036469.2:p.Arg145Ter) (online supplemental table 4). Segregation analysis showed again that the parents are both heterozygotes but the sibling in the intellectual disability category is homozygous for the reference allele. This *CFAP45* variant is in a 8142476 bp ROH (GrCh38 chromosome 1: 158386429–166528905) containing 3821 homozygous and zero heterozygous variants (ROH score 95.53), not present in the sibling.

Next, we searched for other biallelic, potentially causative variants in either *LRRC45* or *CFAP45* across the entire rare disease 100 000 genomes dataset to gain independent replication of causality. No additional potentially pathogenic variants were identified for *CFAP45*. However, we identified a second proband with *LRRC45* variants within the cone-rod dystrophy recruitment category and with an ‘unsolved’ GMC exit questionnaire. We identified a heterozygous *LRRC45* start loss variant: NM_144999.4:c.1A>T, NP_659436.1:p.Met1? (absent from gnomAD, GEL 100K MAF 1.271×10^–5^), and a heterozygous splice acceptor variant: NM_144999.4:c.1126–1G>A (gnomAD allele frequency 8.059×10^–6^, GEL 100K MAF 2.542×10^–5^). The proband was entered as a singleton participant, so parental sequence is not available in the 100,000 Genomes Project or on clinician request to establish phase. *LRRC45* therefore remains a putative novel disease gene accounting for the phenotype in these individuals.

## Discussion

### Diagnosis rate for participants in the CMC cohort of the 100,000 Genomes Project

This study provides a research molecular diagnosis from WGS data for just over half of the participants in the CMC cohort of the 100,000 Genomes Project (43/83, 51.8%), 33 of which are classified as confident or probable (39.8%). Our overall diagnosis rate is 19.3% higher than the 27/83 (32.5%) with GEL reported findings in GMC exit questionnaires (23/83 reported as solved plus 4/83 with VUSs). It is likely that at least nine of the novel research molecular diagnoses would eventually be made and reported by GEL given that they contain only tier 1 or 2 variants (participants #11, #12, #13, #14, #15, #21, #27, #72 and #75). In identifying and alerting clinical teams, we are providing benefit to participants who have, in some cases, been waiting years for identification of a molecular diagnosis (recruitment to the 100,000 Genomes Project ended in 2018).

There are 11 participants with research molecular diagnoses with at least one variant outside of tiers 1 and 2, which would be missed by the standard diagnostic strategy of inspecting only those variants. Therefore, the added diagnostic value of undertaking analyses outside tiers 1 and 2 is at least 11/83 (13.3%). This highlights the value of research collaborations to investigate unsolved cases and improved diagnosis rates from accessible genomic data.

Unfortunately, major challenges remain in returning research identified diagnoses to recruiting clinicians to ensure they are successfully fed back to participants, which is being addressed with collaborators at GEL. Improved communication between recruiting clinicians and researchers would facilitate better interpretation of variants, but a lack of an automated system for researcher/clinician contact introduces a significant bottleneck, and the long time between recruitment and research identified molecular diagnosis has meant that some recruiting clinicians no longer work in the NHS trust and GMC where they recruited patients to the project, and there is no mechanism of forwarding emails in cases such as this. Recruiting clinician collaboration is hugely valuable to provide additional clinical information where required, as well as contacting patients to ask for consent to publication of more detailed clinical data. Furthermore, they can obtain relevant tissue samples to validate variant effects, particularly useful for novel splice variants and SVs.

### Conditions identified

Among probands in the CMC cohort with research molecular diagnoses, a surprisingly high proportion have causative variants in non-ciliopathy genes (19/43, 44.2%). This suggests that there are likely to be significant numbers of participants with ciliopathies recruited to other rare disease categories. This misdiagnosis rate may be because primary ciliopathies can be difficult to recognise clinically due to the great diversity of possible disease features. More specific ‘hard’ phenotypic features can signpost healthcare professionals to the likelihood of a ciliopathy syndrome, but these are not always present. The best example is the molar tooth sign, which is the pathognomonic sign for JBTS-related conditions with no differential diagnoses.^30^ This is reflected in the highest correlation between recruitment category and identified molecular diagnosis rate being for the JBTS group: 6/14 (42.9%) were recruited as suspected JBTS, and then confirmed to have JBTS at the molecular level. Ten of the 14 patients recruited with suspected JBTS had the HPO term ‘Molar Tooth Sign on MRI’ entered by the recruiting clinician, including all six that were solved at the molecular level.

Another reason for the high proportion of non-ciliopathy diagnoses could be limitations or difficulties in choosing appropriate recruitment categories for participants of the 100,000 Genomes Project. Categories may have been selected for convenience or lack of awareness of alternative, potentially more appropriate options. The RMCD category may have been treated as a ‘catch-all’ group for participants with constellations of multisystemic features, not obviously recognisable as a specific syndrome. This is reflected by this group having the lowest diagnosis rate of the three included in the CMC cohort: 9/24 (37.5%) have a research-identified molecular diagnosis, but only two are ciliopathies.

An important outcome to explore further is the relatively high number of participants recruited in the BBS category, found to have variants causative of isolated eye disorders (n=4). It is unclear if recruiting clinicians suspected BBS due to the presence of non-ocular features or whether the participants were inappropriately included in the BBS category. This problem clearly demonstrates the importance of accurate and comprehensive phenotyping to refine the interpretation of sequence variants.

### Mutational mechanism of causative variants

Sixty-four individual, potentially causative variants, have been identified in this research study (online supplemental table 4). Of the variants detected, at least four would not have been detectable or accurately described by WES or gene panel, as they are SVs including significant intronic regions (figure 3). Ideally, all SVs of interest should be confirmed by long-range PCR and either third generation nanopore or Sanger sequencing, but DNA samples from these cases could not be obtained from referring clinicians. A recent study of NHS rare diseases patients undergoing WGS, reported 102 large deletions and six complex SVs from 1103 distinct causal variants (9.8% SVs).^31^ Our identified rate of SVs is slightly lower at 4/64 (6.3%). It seems likely that further SVs are responsible for a proportion of the unsolved participants in the CMC cohort, but strategies to detect them are not yet well established.

WGS, particularly PCR-free WGS, offers great advantages in SV analysis over WES, due to even coverage of the whole genome permitting reliable identification of SVs, but we are yet to fully take advantage of these methodologies. The GEL dataset is being used to improve the way we analyse SVs, with a gnomAD-type database of all SVs in GEL with allele frequencies in the cohort having been developed by Jing Yu in Oxford to permit exclusion of SVs from analysis in a patient if that SV appears above a particular minor allele frequency (MAF) in the GEL dataset. PCR-free WGS adds the further benefit of improved coverage of GC rich regions of the genome that are not efficiently amplified in PCR. As many promoter regions are GC rich, this provides an advantage for identifying regulatory region variants.

A further benefit of WGS over WES or gene panel testing is the opportunity to analyse intronic regions. We used the in silico tool SpliceAI to find variants predicted to cause novel splicing effects and identified three variants outside the canonical splice sites predicted to cause splice donor site defects. No novel splicing variants were identified in genes from the DDG2P gene panel using our SpliceAI script in unsolved participants of the cohort. However, given the diversity of diagnoses, it is highly likely that further causative splicing variants could be found in non-ciliopathy genes. As well as splice variant identification, intronic WGS data can also be interrogated for regulatory region variants implicated in human disease, using resources such as the UTRannotator tool to annotate high-impact 5′ untranslated region variants either creating new upstream opening reading frames (ORFs) or disrupting existing upstream ORFs.^32^

Despite the many advantages of WGS over WES, WES remains a popular sequencing strategy as it involves sequencing of only around 2% of the genome, significantly lowering costs of sequencing, permitting sequencing to greater depth on a limited budget, lowering demands on data storage, increasing analysis times and reducing workload for clinical scientists and researchers to process and interpret the significantly smaller number of identified variants. Furthermore, coding region variants are more straightforward to classify, making analysis of WES data more straightforward than analysis of WGS data.

### Candidate gene analysis

A list of 302 candidate ciliopathy genes (online supplemental table 3) was used in conjunction with our custom variant filtering pipeline in pursuit of diagnosis for probands unsolved through tiered variant analysis. One proband, participant #48, has two homozygous, protein-truncating variants in the candidate ciliopathy genes *LRRC45*, a protein associated with distal appendages of the basal body that contributes to early steps of axoneme extension during ciliogenesis,^33^ and *CFAP45*, a coiled coil domain protein and expressed in nasopharyngeal epithelium and trachea.^34^

There are various possibilities regarding the potential contribution of these variants to the clinical features of proband #48 and their sibling in the intellectual disability category. The two siblings share neurodevelopmental delay and intellectual disability. Proband #48 also has additional features in keeping with both syndromic primary and motile ciliopathies. *CFAP45* has been recently published as a motile ciliopathy gene,^35^ so it is possible that the homozygous nonsense *CFAP45* variant present in participant #48 but not their sibling could account for the clinical motile ciliopathy features in participant #48, with the *LRRC45* variants accounting for the neurodevelopmental delay and intellectual disability in both siblings.

Given the phenotypic heterogeneity in ciliopathies even within families with the same variant, another hypothesis is that the two siblings have different presentations of a condition caused by their shared homozygous *LRRC45* frameshift variant. The putative loss of function (pLoF) gnomAD score for *LRRC45* (pLoF=0.88) suggests that *LRRC45* is not tolerant to loss of function.^28^ The additional proband from the cone-rod dystrophy category with compound heterozygous high impact *LRRC45* variants adds to the evidence that this may be a ciliopathy gene.

### Value of diagnoses

Undertaking broad genomic tests like WES and WGS can curtail the ‘diagnostic odyssey’ experienced by many patients with rare disorders, potentially sparing them multiple invasive tests and misdiagnoses.^36^ Analysis can be iterative such that the data can be ‘opened up’ beyond the first virtual gene panel without the need for serial testing. Results from this study demonstrate the value of this approach, given the high proportion of participants with non-ciliopathy diagnoses. The NHS Genomic Medicine service, introduced in 2018 as a follow on from the 100,000 Genomes Project, provides a curated National Genomic Test Directory including WES and WGS where appropriate.^20^ This will embed genomic testing into mainstream care and standardise testing across the country.

Determining the underlying genotype for a patient’s phenotype allows provision of accurate information about their condition, including potential current and future associated features for which screening or treatment may be available. An example of this in action is participant #61, recruited in the RMCD category. An untiered heterozygous missense variant in *WT1* was identified through our filtering that is listed as pathogenic on ClinVar, in keeping with autosomal dominant WT1-related disorder. This diagnosis, which was successfully fed back to the recruiting clinician, is considered especially important given the associated risk of Wilms’ tumour and the recommendation for regular screening to facilitate early detection and treatment.^37^

Lack of a genetic diagnosis can lead to inappropriate management of conditions and delays in accessing specialised services such as the multidisciplinary service for BBS and Alström syndrome in Birmingham Children’s Hospital and Great Ormond Street Hospital in the UK. Without greater awareness and higher diagnosis rates of ciliopathies, it may continue to be difficult to secure funding for additional specialist services for rare ciliopathies.

### Perspective on the future of genetic diagnosis

This study prompts reconsideration of approaches to genetic diagnostics, particularly traditional forward genetics in comparison with reverse phenotyping. Classically, clinicians have suggested a possible underlying diagnosis based on the collection of clinical features observed, then the lab have tested for variants in gene(s) associated with that suspected diagnosis. This study demonstrates the utility of a reverse genetics strategy, by going ‘backwards’ from variants that are assessed as pathogenic at the molecular level, to determine if they could match with the patient’s features and the disease’s inheritance pattern. As the cost and availability of large-scale sequencing tests including WES and WGS continues to fall, this reverse phenotyping strategy is becoming increasingly integrated into NHS genetic diagnostics. With this, the current bottleneck is clinical interpretation of variants. To realise the potential of WES and WGS, investment into dedicated time and resourcing for specialist variant interpretation is essential, as is careful and comprehensive phenotyping and strong communication between clinical scientists, clinical geneticists, mainstream clinicians and researchers. Improved integration of SV and splice variant analysis tools, such as SpliceAI, will be essential to maximise the diagnostic potential of WGS data beyond coding variants in exons of virtual panels of genes. The 19.3% genetic diagnosis uplift achieved in our study demonstrates what can be achieved with additional time and resources invested into WGS analysis. Now that this variant filtering and analysis pipeline has been established, we anticipate that this additional analysis can be achieved within days or weeks rather than months.

Clearly, large-scale genomic studies such as the 100,000 Genomes Project offer huge opportunities to improve diagnostics, understanding of disease mechanisms and identification of novel drug targets. The current challenge is to improve our strategies to analyse sequence data to provide the maximum benefit for patients and the scientific community.

## Data Availability

Data may be obtained from a third party and are not publicly available. Full data is available in the Secure Genomic England Secure Research Environment.
