# Supplementary material for: Molecular diagnoses in the congenital malformations caused by ciliopathies cohort of the 100,000 Genomes Project
Source: J Med Genet. 2021 Oct 29;59(8):737–47. doi: 10.1136/jmedgenet-2021-108065 (PMC9340050; doi:10.1136/jmedgenet-2021-108065)
Supplement: Supplementary data [file jmedgenet-2021-108065supp001.pdf]

Supplementary tables

Supplementary Table 1. Summary of genetic diagnosis rates of various ciliopathies using different next generation sequencing approaches.

Patient group	Sequencing approach	Genetic diagnosis rate	Year	Reference
10 families with NPHP	homozygosity mapping + WES	70%	2014	(1)
Syndromic ciliopathies	Gene panel, SNP genotyping, targeted sequencing of candidate genes	62%	2015	(2)
375 families with JBTS	Gene panel	32%	2015	(3)
Ciliopathy patients in a large rare disease cohort	WES	44%	2015	(4)
79 suspected NPHP cases	WES	40.5%	2016	(5)
43 patients with NPHP who had had the 5 most common NPHP genes excluded as a cause of their disease	Gene panel	16.3%	2016	(6)
26 patients with JBTS or MKS	WES with split read mapping	46%	2016	(7)
6 BBS patients	WES	67%	2017	(8)
100 families with JBTS	Gene panel sequencing + WES	94%	2017	(9)
56 families in whom PCD was suspected	WES	68%	2020	(10)
125 families with ciliopathies	WGS	87%	2020	(11)
29 families with skeletal ciliopathies	WGS + structural variant screening + RNA analyses	90%	2021	(12)

Supplementary Table 2. Diagnostic grade "green" genes in Rare Multisystem Ciliopathy Disorders PanelApp panel V1.139

Gene name	Mode of inheritance	Associated phenotypes	Ensembl Id
AHI1	Biallelic	JBTS	ENSG00000135541
ALMS1	Biallelic	BBS, Alström	ENSG00000116127
ANKS6	Biallelic	PKD, NPHP	ENSG00000165138
ARL13B	Biallelic	JBTS	ENSG00000169379
ARL6	Biallelic	BBS	ENSG00000113966
ARMC9	Biallelic	JBTS	ENSG00000135931
B9D2	Biallelic	JBTS, MKS	ENSG00000123810
BBS1	Biallelic	BBS	ENSG00000174483
BBS10	Biallelic	BBS	ENSG00000179941

BBS12	Biallelic	BBS	ENSG00000181004
BBS2	Biallelic	BBS	ENSG00000125124
BBS4	Biallelic	BBS	ENSG00000140463
BBS5	Biallelic	BBS	ENSG00000163093
BBS7	Biallelic	BBS	ENSG00000138686
BBS9	Biallelic	BBS	ENSG00000122507
C21orf2	Biallelic	JATD, Spondylometaphyseal dysplasia, IRD	ENSG00000160226
C2CD3	Biallelic	SRPS, JATD, OFD	ENSG00000168014
C5orf42	Biallelic	JBTS, OFD	ENSG00000197603
CC2D2A	Biallelic	JBTS, MKS, COACH	ENSG00000048342
CENPF	Biallelic	Stromme	ENSG00000117724
CEP104	Biallelic	JBTS	ENSG00000116198
CEP120	Biallelic	SRTD, CED, JATD	ENSG00000168944
CEP164	Biallelic	NPHP, SLS	ENSG00000110274
CEP290	Biallelic	JBTS, MKS, COACH, SLS	ENSG00000198707
CEP41	Biallelic	JBTS	ENSG00000106477
CEP83	Biallelic	NPHP	ENSG00000173588
CRB2	Biallelic	PKD with ventriculomegaly	ENSG00000148204
CSPP1	Biallelic	JBTS, MKS	ENSG00000104218
DDX59	Biallelic	OFD	ENSG00000118197
DHCR7	Biallelic	SLO	ENSG00000172893
DYNC2H1	Biallelic	SRTD, CED, JATD	ENSG00000187240
DYNC2LI1	Biallelic	SRTD	ENSG00000138036
EVC	Biallelic	EVC, WAD	ENSG00000072840
EVC2	Biallelic	EVC, WAD	ENSG00000173040
GLI3	Monoallelic	JBTS, SLS	ENSG00000106571
HNF1B	Monoallelic	PKD, NPHP	ENSG00000275410
HYLS1	Biallelic	JBTS, Hydrolethalus syndrome	ENSG00000198331
ICK	Biallelic	Endocrine-cerebro-osteodysplasia	ENSG00000112144
IFT122	Biallelic	CED	ENSG00000163913
IFT140	Biallelic	SRTD, JATD, Mainzer-Saldino	ENSG00000187535
IFT172	Biallelic	RP, SRTD, JATD, Mainzer-Saldino, SRTD	ENSG00000138002
IFT27	Biallelic	? BBS	ENSG00000100360
IFT43	Biallelic	SRTD, CED, Sensenbrennar syndrome	ENSG00000119650
IFT52	Biallelic	SRTD	ENSG00000101052
IFT74	Biallelic	? BBS	ENSG00000096872
IFT80	Biallelic	SRTD, JATD	ENSG00000068885
INPP5E	Biallelic	JBTS	ENSG00000148384
INVS	Biallelic	NPHP, SLS	ENSG00000119509
IQCB1	Biallelic	SLS	ENSG00000173226

KIAA0586	Biallelic	JBTS, SRTD	ENSG00000100578
KIAA0753	Biallelic	OFD, SRTD, JBTS	ENSG00000198920
KIF7	Biallelic	JBTS, Acrocallosal syndrome	ENSG00000166813
LZTFL1	Biallelic	BBS	ENSG00000163818
MAPKBP1	Biallelic	NPHP	ENSG00000137802
MKKS	Biallelic	BBS	ENSG00000125863
MKS1	Biallelic	MKS, BBS, JBTS	ENSG00000011143
NEK1	Biallelic	SRTD	ENSG00000137601
NEK8	Biallelic	NPHP	ENSG00000160602
NPHP1	Biallelic	NPHP, JBTS, SLS	ENSG00000144061
NPHP3	Biallelic	MKS, SLS, NPHP	ENSG00000113971
NPHP4	Biallelic	NPHP, SLS	ENSG00000131697
OFD1	X-linked	JBTS, OFD	ENSG00000046651
PIBF1	Biallelic	JBTS	ENSG00000083535
PKD1	Monoallelic and biallelic	PKD	ENSG00000008710
PKD2	Monoallelic	PKD	ENSG00000118762
PKHD1	Biallelic	Polycystic kidney and hepatic disease	ENSG00000170927
PMM2	Biallelic	Congenital disorder of glycosylation	ENSG00000140650
RPGRIP1L	Biallelic	JBTS, MKS	ENSG00000103494
SBDS	Biallelic	Skeletal ciliopathies	ENSG00000126524
SCLT1	Biallelic	OFD, SLS	ENSG00000151466
SDCCAG8	Biallelic	BBS, SLS	ENSG00000054282
TCTEX1D2	Biallelic	SRTD, JATD	ENSG00000213123
TCTN1	Biallelic	JBTS	ENSG00000204852
TCTN2	Biallelic	JBTS, MKS	ENSG00000168778
TCTN3	Biallelic	OFD, JBTS, MKS, Mohr-Majewski syndrome	ENSG00000119977
TMEM107	Biallelic	MKS, OFD, ? JBTS	ENSG00000179029
TMEM138	Biallelic	JBTS	ENSG00000149483
TMEM216	Biallelic	JBTS, MKS	ENSG00000187049
TMEM231	Biallelic	JBTS, MKS	ENSG00000205084
TMEM237	Biallelic	JBTS	ENSG00000155755
TMEM67	Biallelic	JBTS, MKS, COACH, NPHP, Senior-Boichis syndrome, ? BBS	ENSG00000164953
TRAF3IP1	Biallelic	SLS	ENSG00000204104
TTC21B	Biallelic	NPHP, SRTD, JATD	ENSG00000123607
TTC8	Biallelic	BBS	ENSG00000165533
TXNDC15	Biallelic	MKS	ENSG00000113621
VPS13B	Biallelic	Cohen syndrome	ENSG00000132549
WDPCP	Biallelic	MKS, ?BBS	ENSG00000143951
WDR19	Biallelic	CED, SRTD, JATD, NPHP, SLS	ENSG00000157796

WDR34	Biallelic	SRTD, JATD	ENSG00000119333
WDR35	Biallelic	CED, SRTD	ENSG00000118965
WDR60	Biallelic	SRTD, JATD	ENSG00000126870
ZSWIM6	Monoallelic	Acromelic frontonasal dysostosis	ENSG00000130449
ISCA-37405-Loss	Biallelic	JBTS, NPHP	
ISCA-37432-Loss	Monoallelic	Renal cysts and diabetes syndrome, Autism Spectrum Disorder, Mayer-Rokitansky-Kuster-Hauser syndrome	

Abbreviations: BBS: Bardet Biedl syndrome; CED: Cranioectodermal dysplasia; COACH: cerebellar vermis hypo/aplasia, oligophrenia, ataxia, ocular coloboma, and hepatic fibrosis; EVC: Ellis-Van-Creveld syndrome; IRD: Inherited retinal dystrophy; JATD: Jeune asphyxiating thoracic dystrophy; JBTS: Joubert syndrome; LCA: Leber's congenital amaurosis; MKS: Meckel Gruber Syndrome; NPHP: nephronophthisis; PKD: Polycystic kidney disease; RP: Retinitis Pigmentosa; SLO: Smith Lemli Optiz syndrome; SLS: Senior Loken syndrome; SRPS: Short rib polydactyly syndrome; SRTD: Short rib thoracic dystrophy; WAD: Weyers acrofacial dysostosis

Supplementary Table 2. Candidate gene list provided alongside Rare Multisystem Super Ciliopathy panel v4.91 for Extract_hets Python script.

Gene symbol	Source	Ensembl Id
ACVR2B	PanelApp RMCD red gene	ENSG00000114739
ADAMTS10	Ciliopathy candidate identified through local research team experimental screen(s)	ENSG00000142303
ADAMTS9	Ciliopathy candidate identified through local research team experimental screen(s)	ENSG00000163638
ADCY3	On SCGS V1 list	ENSG00000138031
ADGRV1	PanelApp RMCD red gene + SCGS V1 list	ENSG00000164199
AIPL1	PanelApp RMCD red gene + SCGS V1 list	ENSG00000129221
AK7	On SCGS V1 list	ENSG00000140057
AK8	On SCGS V1 list	ENSG00000165695
ARF4	On SCGS V1 list	ENSG00000168374
ARL3	On SCGS V1 list	ENSG00000138175
ARMC4	PanelApp RMCD red gene	ENSG00000169126
ASAP1	On SCGS V1 list	ENSG00000153317
ATXN10	PanelApp RMCD red gene + SCGS V1 list	ENSG00000130638
B9D1	PanelApp RMCD red gene + SCGS V1 list	ENSG00000108641
BBIP1	PanelApp RMCD red gene	ENSG00000214413
C21orf59	PanelApp RMCD red gene	ENSG00000159079
C2orf71	PanelApp RMCD red gene	ENSG00000179270
C8orf37	PanelApp RMCD red gene + SCGS V1 list	ENSG00000156172

CBY1	On SCGS V1 list	ENSG00000100211
CCDC103	PanelApp RMCD red gene + SCGS V1 list	ENSG00000167131
CCDC114	PanelApp RMCD red gene + SCGS V1 list	ENSG00000105479
CCDC151	PanelApp RMCD red gene	ENSG00000198003
CCDC28B	PanelApp RMCD red gene + SCGS V1 list	ENSG00000160050
CCDC39	PanelApp RMCD red gene + SCGS V1 list	ENSG00000145075
CCDC40	PanelApp RMCD red gene + SCGS V1 list	ENSG00000141519
CCDC65	PanelApp RMCD red gene	ENSG00000139537
CCNO	PanelApp RMCD red gene	ENSG00000152669
CCP110	On SCGS V1 list	ENSG00000103540
CDH23	PanelApp RMCD red gene + SCGS V1 list	ENSG00000107736
CENPJ	On SCGS V1 list	ENSG00000151849
CEP131	On SCGS V1 list	ENSG00000141577
CEP135	On SCGS V1 list	ENSG00000174799
CEP250	On SCGS V1 list	ENSG00000126001
CEP72	On SCGS V1 list	ENSG00000112877
CEP89	On SCGS V1 list	ENSG00000121289
CEP97	On SCGS V1 list	ENSG00000182504
CFAP100	On SCGS V1 list	ENSG00000163885
CFAP43	PanelApp RMCD red gene	ENSG00000197748
CFAP44	PanelApp RMCD red gene	ENSG00000206530
CFAP45	Ciliopathy candidate identified through local research team experimental screen(s)	ENSG00000213085
CFAP53	PanelApp RMCD red gene	ENSG00000172361
CFAP57	Ciliopathy candidate identified through local research team experimental screen(s)	ENSG00000243710
CFC1	PanelApp RMCD red gene	ENSG00000136698
CFTR	PanelApp RMCD red gene	ENSG00000001626
CLDN2	On SCGS V1 list	ENSG00000165376
CLRN1	PanelApp RMCD red gene	ENSG00000163646
CLTB	Ciliopathy candidate identified through local research team experimental screen(s)	ENSG00000175416
CLUAP1	On SCGS V1 list	ENSG00000103351
CNGA2	On SCGS V1 list	ENSG00000183862
CNGA4	On SCGS V1 list	ENSG00000132259
CNGB1	On SCGS V1 list	ENSG00000070729
CRB1	PanelApp RMCD red gene	ENSG00000134376
CRB3	On SCGS V1 list	ENSG00000130545
CRELD1	PanelApp RMCD red gene	ENSG00000163703
CROCC	On SCGS V1 list	ENSG00000058453
CRX	PanelApp RMCD red gene	ENSG00000105392
CTNNB1	On SCGS V1 list	ENSG00000168036

DCDC2	PanelApp RMCD red gene + SCGS V1 list	ENSG00000146038
DISC1	On SCGS V1 list	ENSG00000162946
DNAAF1	PanelApp RMCD red gene + SCGS V1 list	ENSG00000154099
DNAAF2	PanelApp RMCD red gene + SCGS V1 list	ENSG00000165506
DNAAF3	PanelApp RMCD red gene + SCGS V1 list	ENSG00000167646
DNAAF4	PanelApp RMCD red gene	ENSG00000256061
DNAAF5	PanelApp RMCD red gene	ENSG00000164818
DNAH1	PanelApp RMCD red gene + SCGS V1 list	ENSG00000114841
DNAH10	On SCGS V1 list	ENSG00000197653
DNAH11	PanelApp RMCD red gene + SCGS V1 list	ENSG00000105877
DNAH2	On SCGS V1 list	ENSG00000183914
DNAH5	PanelApp RMCD red gene + SCGS V1 list	ENSG00000039139
DNAH6	On SCGS V1 list	ENSG00000115423
DNAI1	PanelApp RMCD red gene + SCGS V1 list	ENSG00000122735
DNAI2	PanelApp RMCD red gene + SCGS V1 list	ENSG00000171595
DNAJB13	PanelApp RMCD red gene	ENSG00000187726
DNAL1	PanelApp RMCD red gene + SCGS V1 list	ENSG00000119661
DNALI1	On SCGS V1 list	ENSG00000163879
DNHD1	PanelApp RMCD red gene	ENSG00000179532
DPCD	On SCGS V1 list	ENSG00000166171
DPYSL2	On SCGS V1 list	ENSG00000092964
DRC1	PanelApp RMCD red gene + SCGS V1 list	ENSG00000157856
DRD1	On SCGS V1 list	ENSG00000184845
DRD2	On SCGS V1 list	ENSG00000149295
DRD5	On SCGS V1 list	ENSG00000169676
DVL1	On SCGS V1 list	ENSG00000107404
DYNLT1	On SCGS V1 list	ENSG00000146425
EFHC1	On SCGS V1 list	ENSG00000096093
EXOC3	On SCGS V1 list	ENSG00000180104
EXOC3L2	PanelApp RMCD amber gene	ENSG00000283632
EXOC4	On SCGS V1 list	ENSG00000131558
EXOC5	On SCGS V1 list	ENSG00000070367
EXOC6	On SCGS V1 list	ENSG00000138190
EXOC6B	On SCGS V1 list	ENSG00000144036
EXOC8	PanelApp RMCD red gene	ENSG00000116903
EZH2	On SCGS V1 list	ENSG00000106462
FAM149B1	PanelApp RMCD amber gene	ENSG00000138286
FAM161A	On SCGS V1 list	ENSG00000170264
FBF1	On SCGS V1 list	ENSG00000188878
FLNA	On SCGS V1 list	ENSG00000196924
FOPNL	On SCGS V1 list	ENSG00000133393

FOXH1	PanelApp RMCD red gene	ENSG00000160973
FOXJ1	On SCGS V1 list	ENSG00000129654
FUZ	On SCGS V1 list	ENSG00000010361
GAS8	PanelApp RMCD red gene + SCGS V1 list	ENSG00000141013
GDF1	PanelApp RMCD red gene	ENSG00000130283
GLI1	On SCGS V1 list	ENSG00000111087
GLI2	On SCGS V1 list	ENSG00000074047
GLIS2	PanelApp RMCD red gene + SCGS V1 list	ENSG00000126603
GPR161	On SCGS V1 list	ENSG00000143147
GSK3B	On SCGS V1 list	ENSG00000082701
GUCY2D	PanelApp RMCD red gene	ENSG00000132518
HAP1	On SCGS V1 list	ENSG00000173805
HSD11B1	On SCGS V1 list	ENSG00000117594
HSPA8	On SCGS V1 list	ENSG00000109971
HSPB11	On SCGS V1 list	ENSG00000081870
HTR6	On SCGS V1 list	ENSG00000158748
HTT	On SCGS V1 list	ENSG00000197386
HYDIN	PanelApp RMCD red gene + SCGS V1 list	ENSG00000157423
IFT122	On SCGS V1 list	ENSG00000128581
IFT20	On SCGS V1 list	ENSG00000109083
IFT46	On SCGS V1 list	ENSG00000118096
IFT57	On SCGS V1 list	ENSG00000114446
IFT81	PanelApp RMCD red gene + SCGS V1 list	ENSG00000122970
IFT88	On SCGS V1 list	ENSG00000032742
IMPDH1	PanelApp RMCD red gene	ENSG00000106348
INTU	On SCGS V1 list	ENSG00000164066
JADE1	On SCGS V1 list	ENSG00000077684
KCNJ13	PanelApp RMCD red gene	ENSG00000115474
KIAA0556	PanelApp RMCD red gene	ENSG00000047578
KIF14	PanelApp RMCD red gene	ENSG00000118193
KIF17	On SCGS V1 list	ENSG00000117245
KIF19	On SCGS V1 list	ENSG00000196169
KIF24	On SCGS V1 list	ENSG00000186638
KIF27	On SCGS V1 list	ENSG00000165115
KIF3A	On SCGS V1 list	ENSG00000131437
KIF3B	On SCGS V1 list	ENSG00000101350
KIF3C	On SCGS V1 list	ENSG00000084731
KIF5B	On SCGS V1 list	ENSG00000170759
LBR	PanelApp RMCD red gene	ENSG00000143815
LCA5	PanelApp RMCD red gene + SCGS V1 list	ENSG00000135338
LEFTY2	PanelApp RMCD red gene	ENSG00000143768
LRAT	PanelApp RMCD red gene	ENSG00000121207

LRRC6	PanelApp RMCD red gene + SCGS V1 list	ENSG00000129295
LRRC45	Ciliopathy candidate identified through local research team experimental screen(s)	ENSG00000169683
MAK	On SCGS V1 list	ENSG00000111837
MAL	On SCGS V1 list	ENSG00000172005
MAPRE1	On SCGS V1 list	ENSG00000101367
MCHR1	On SCGS V1 list	ENSG00000128285
MCIDAS	PanelApp RMCD red gene	ENSG00000234602
MDM1	On SCGS V1 list	ENSG00000111554
MICAL2	Ciliopathy candidate identified through local research team experimental screen(s)	ENSG00000133816
MLF1	On SCGS V1 list	ENSG00000178053
MNS1	On SCGS V1 list	ENSG00000138587
MUC1	PanelApp RMCD red gene	ENSG00000185499
MYO15A	On SCGS V1 list	ENSG00000091536
MYO7A	PanelApp RMCD red gene + SCGS V1 list	ENSG00000137474
NEK2	On SCGS V1 list	ENSG00000117650
NEK4	On SCGS V1 list	ENSG00000114904
NGFR	On SCGS V1 list	ENSG00000064300
NIN	On SCGS V1 list	ENSG00000100503
NINL	On SCGS V1 list	ENSG00000101004
NKX2-5	PanelApp RMCD red gene	ENSG00000183072
NME5	On SCGS V1 list	ENSG00000112981
NME7	On SCGS V1 list	ENSG00000143156
NME8	PanelApp RMCD red gene + SCGS V1 list	ENSG00000086288
NODAL	PanelApp RMCD red gene	ENSG00000156574
NOTO	On SCGS V1 list	ENSG00000214513
NUP214	On SCGS V1 list	ENSG00000126883
NUP35	On SCGS V1 list	ENSG00000163002
NUP37	On SCGS V1 list	ENSG00000075188
NUP62	On SCGS V1 list	ENSG00000213024
NUP93	On SCGS V1 list	ENSG00000102900
OCRL	PanelApp RMCD red gene + SCGS V1 list	ENSG00000122126
ODF2	On SCGS V1 list	ENSG00000136811
ORC1	On SCGS V1 list	ENSG00000085840
PACRG	On SCGS V1 list	ENSG00000112530
PAFAH1B1	On SCGS V1 list	ENSG00000007168
PARD3	On SCGS V1 list	ENSG00000148498
PARD6A	On SCGS V1 list	ENSG00000102981
PCDH15	PanelApp RMCD red gene + SCGS V1 list	ENSG00000150275
PCM1	On SCGS V1 list	ENSG00000078674
PDE6D	PanelApp RMCD red gene + SCGS V1 list	ENSG00000156973

PDZD7	On SCGS V1 list	ENSG00000186862
PKD1L1	On SCGS V1 list	ENSG00000158683
PLK1	On SCGS V1 list	ENSG00000166851
POC1A	PanelApp RMCD red gene + SCGS V1 list	ENSG00000164087
POC1B	PanelApp RMCD amber gene	ENSG00000139323
PPP3CC	Ciliopathy candidate identified through local research team experimental screen(s)	ENSG00000120910
PRKCSH	PanelApp RMCD red gene	ENSG00000130175
PTCH1	On SCGS V1 list	ENSG00000185920
PTPDC1	On SCGS V1 list	ENSG00000158079
RAB11A	On SCGS V1 list	ENSG00000103769
RAB11FIP3	On SCGS V1 list	ENSG00000090565
RAB17	On SCGS V1 list	ENSG00000124839
RAB23	On SCGS V1 list	ENSG00000112210
RAB3IP	On SCGS V1 list	ENSG00000127328
RAB8A	On SCGS V1 list	ENSG00000167461
RAN	On SCGS V1 list	ENSG00000132341
RANBP1	On SCGS V1 list	ENSG00000099901
RD3	PanelApp RMCD red gene	ENSG00000198570
RDH12	PanelApp RMCD red gene	ENSG00000139988
RFX3	On SCGS V1 list	ENSG00000080298
RILPL1	On SCGS V1 list	ENSG00000188026
RILPL2	On SCGS V1 list	ENSG00000150977
ROPN1L	On SCGS V1 list	ENSG00000145491
RP1	On SCGS V1 list	ENSG00000104237
RP2	On SCGS V1 list	ENSG00000102218
RPE65	PanelApp RMCD red gene	ENSG00000116745
RPGR	PanelApp RMCD red gene + SCGS V1 list	ENSG00000156313
RPGRIP1	PanelApp RMCD red gene + SCGS V1 list	ENSG00000092200
RSPH1	PanelApp RMCD red gene + SCGS V1 list	ENSG00000160188
RSPH3	On SCGS V1 list	ENSG00000130363
RSPH4A	PanelApp RMCD red gene + SCGS V1 list	ENSG00000111834
RSPH9	PanelApp RMCD red gene + SCGS V1 list	ENSG00000172426
RTTN	On SCGS V1 list	ENSG00000176225
SASS6	On SCGS V1 list	ENSG00000156876
SCNN1A	PanelApp RMCD red gene	ENSG00000111319
SCNN1B	PanelApp RMCD red gene	ENSG00000168447
SCNN1G	PanelApp RMCD red gene	ENSG00000166828
SEC63	PanelApp RMCD red gene	ENSG00000025796
SEPT2	On SCGS V1 list	ENSG00000168385
SEPT7	On SCGS V1 list	ENSG00000122545
SHH	On SCGS V1 list	ENSG00000164690

SLC47A2	On SCGS V1 list	ENSG00000180638
SMCR8	Ciliopathy candidate identified through local research team experimental screen(s)	ENSG00000283741
SMO	On SCGS V1 list	ENSG00000128602
SNAP25	On SCGS V1 list	ENSG00000132639
SNX10	On SCGS V1 list	ENSG00000086300
SNX17	On SCGS V1 list	ENSG00000115234
SPA17	On SCGS V1 list	ENSG00000064199
SPAG1	PanelApp RMCD red gene	ENSG00000104450
SPAG16	On SCGS V1 list	ENSG00000144451
SPAG17	On SCGS V1 list	ENSG00000155761
SPAG6	On SCGS V1 list	ENSG00000077327
SPATA7	PanelApp RMCD red gene + SCGS V1 list	ENSG00000042317
SPEF2	On SCGS V1 list	ENSG00000152582
SPTBN4	Ciliopathy candidate identified through local research team experimental screen(s)	ENSG00000160460
SSNA1	On SCGS V1 list	ENSG00000176101
SSTR3	On SCGS V1 list	ENSG00000278195
STIL	On SCGS V1 list	ENSG00000123473
STK36	On SCGS V1 list	ENSG00000163482
STK38L	On SCGS V1 list	ENSG00000211455
STOML3	On SCGS V1 list	ENSG00000133115
STX3	On SCGS V1 list	ENSG00000166900
SUFU	PanelApp RMCD amber gene + SCGS V1 list	ENSG00000107882
SYNE2	On SCGS V1 list	ENSG00000054654
TAPT1	PanelApp RMCD red gene	ENSG00000169762
TBC1D30	On SCGS V1 list	ENSG00000111490
TBC1D32	PanelApp RMCD red gene	ENSG00000146350
TBC1D7	On SCGS V1 list	ENSG00000145979
TEKT2	On SCGS V1 list	ENSG00000092850
TEKT4	On SCGS V1 list	ENSG00000163060
TEKT5	On SCGS V1 list	ENSG00000153060
TEX12	Ciliopathy candidate identified through local research team experimental screen(s)	ENSG00000150783
TNPO1	On SCGS V1 list	ENSG00000083312
TOPORS	PanelApp RMCD red gene + SCGS V1 list	ENSG00000197579
TPPP2	On SCGS V1 list	ENSG00000179636
TRAPPC10	On SCGS V1 list	ENSG00000160218
TRAPPC3	On SCGS V1 list	ENSG00000054116
TRAPPC9	On SCGS V1 list	ENSG00000167632
TRIM32	PanelApp RMCD red gene + SCGS V1 list	ENSG00000119401
TRIP11	On SCGS V1 list	ENSG00000100815

TSC1	PanelApp RMCD red gene	ENSG00000165699
TSC2	PanelApp RMCD red gene	ENSG00000103197
TTBK2	PanelApp RMCD red gene + SCGS V1 list	ENSG00000128881
TTC12	On SCGS V1 list	ENSG00000149292
TTC26	On SCGS V1 list	ENSG00000105948
TTC29	On SCGS V1 list	ENSG00000137473
TTC30A	On SCGS V1 list	ENSG00000197557
TTC30B	On SCGS V1 list	ENSG00000196659
TTK	On SCGS V1 list	ENSG00000112742
TTLL3	On SCGS V1 list	ENSG00000214021
TTLL6	On SCGS V1 list	ENSG00000170703
TTLL9	On SCGS V1 list	ENSG00000131044
TUBA1A	On SCGS V1 list	ENSG00000167552
TUBA1C	On SCGS V1 list	ENSG00000167553
TUBA4A	On SCGS V1 list	ENSG00000127824
TUBB2A	On SCGS V1 list	ENSG00000137267
TUBB2B	On SCGS V1 list	ENSG00000137285
TUBB3	On SCGS V1 list	ENSG00000258947
TUBB8	Ciliopathy candidate identified through local research team experimental screen(s)	ENSG00000261456
TUBE1	On SCGS V1 list	ENSG00000074935
TUBGCP2	On SCGS V1 list	ENSG00000130640
TUBGCP3	On SCGS V1 list	ENSG00000126216
TUBGCP4	On SCGS V1 list	ENSG00000137822
TUBGCP5	On SCGS V1 list	ENSG00000275835
TUBGCP6	On SCGS V1 list	ENSG00000128159
TULP1	PanelApp RMCD red gene + SCGS V1 list	ENSG00000112041
TULP3	On SCGS V1 list	ENSG00000078246
ULK4	On SCGS V1 list	ENSG00000168038
UMOD	PanelApp RMCD red gene	ENSG00000169344
USH1C	PanelApp RMCD red gene + SCGS V1 list	ENSG00000006611
USH1G	PanelApp RMCD red gene + SCGS V1 list	ENSG00000182040
USH2A	PanelApp RMCD red gene + SCGS V1 list	ENSG00000042781
VANGL2	On SCGS V1 list	ENSG00000162738
VDAC3	On SCGS V1 list	ENSG00000078668
VHL	PanelApp RMCD red gene + SCGS V1 list	ENSG00000134086
WDR63	PanelApp RMCD red gene	ENSG00000162643
WDR78	On SCGS V1 list	ENSG00000152763
WHRN	PanelApp RMCD red gene + SCGS V1 list	ENSG00000095397
XPNPEP3	PanelApp RMCD red gene + SCGS V1 list	ENSG00000196236
ZIC3	PanelApp RMCD red gene	ENSG00000156925
ZMYND10	PanelApp RMCD red gene	ENSG00000004838

ZNF423	PanelApp RMCD amber gene + SCGS V1 list	ENSG00000102935

Abbreviations: RMCD = Rare multisystem Ciliopathy disorders, SCGS V1 = SYSCILIA gold standard list version 1

Supplementary Table 4. Detailed variant information for all variants identified amongst participants of the congenital malformations caused by ciliopathies cohort with research identified diagnoses.

Proband research number	Gene symbol	HGVSc	HGVSp	Segregation	Zygosity (presumed where segregation data unk)	Mutational mechanism	SIFT	PolyPhen	1000G AF	gnomAD AF	ClinVar	PMID	CADD Phred	SpliceAI max DS	Match for phenotype	Variant ACMG class
1	*CHD7*	NM_017780.4:c.6955C>T	NP_060250.2:p.Arg2319Cys	De Novo	Het	Mis	Delet (0)	Prob_dam (0.99)	-	-	Path	16400610	27.1		Full	5
2	*ALMS1*	NM_001378454.1:c.10772del	NP_001365383.1:p.Thr3591LysfsTer6	Pat	Comp het	SG	-	-	-	0.00005227	Path	11941369, 11941370, 17594715	23		Full	5
2	*ALMS1*	NM_001378454.1:c.11104C>T	NP_001365383.1:p.Arg3702Ter	Mat	Comp het	SG	-	-	-	0.00001205	Path	-	34		Full	5
3	*ARL6*	NM_001278293.3:c.534A>G	NP_001265222.1:p.Gln178=	Bi-parental	Hom	Syn	-	-	-	0.00000796	-	27708425	24.1	DS_DL 0.87	Full	5
3	*IMPG2*	NM_016247.4:c.3262C>T	NP_057331.2:p.Arg1088Ter	Bi-parental	Hom	SG	-	-	0.0002	0.0000358	Path, Lik_Path	-	42		Partial	4
4	*RPGR*	NM_000328.3:c.1627del	NP_000319.1:p.Asp543IlefsTer11	Unk	XLR	FS, male	-	-	-	-	-	-	-		Partial	4
5	*CEP290*	NM_025114.4:c.5668G>T	NP_079390.3:p.Gly1890Ter	Unk	Comp het	SG	-	-	0.0002	0.00009486	Path	18414213, 26092869, 16682970, 16682973, 17564967	35		Full	5
5	*CEP290*	NM_025114.4:c.322C>T	NP_079390.3:p.Arg108Ter	Unk	Comp het	SG	-	-	-	0.00000402	Path	-	35		Full	5
6	*KIAA0586*	NM_001329943.3:c.392del	NP_001316872.1:p.Arg131LysfsTer4	Pat	Comp het	FS	-	-	0.0024	0.00305	Path, Lik_Path	25741868, 26096313, 28552196, 26386247	33		Full	5
6	*KIAA0586*	NM_001329943.3:c.1402_1408del	NP_001316872.1:p.Pro468IlefsTer15	Mat	Comp het	FS	-	-	-	-	-	-	-		Full	5
7	*CRYBB1*	NM_001887.4:c.193G>A	NP_001878.1:p.Glu65Lys	Unk	Het	Mis	Delet (0)	Prob_dam (0.979)	-	-	-	-	29.2		Partial	3
7	*OFD1*	NM_003611.3:c.306del	NP_003602.1:p.Glu103LysfsTer42	Unk	XLD	FS, female	-	-	-	-	-	-	-		Full	4

7	*PKD1*	NM_001009944.3:c.5890C>T	NP_001009944.3:p.Arg1964Cys	Unk	Het	Mis	Delet (0.03)	poss_dam (0.772)	-	0.00001316	-	-	25.5		Partial	3
8	*PRPF8*	NM_006445.4:c.5804G>A	NP_006436.3:p.Arg1935His	De Novo	Het	Mis	Delet (0)	Prob_dam (1)	-	-	Path, Lik_Path	-	28.3		Full	4
9	*CEP152*	NM_001194998.2:c.2041C>T	NP_001181927.1:p.His681Tyr	Pat	Comp het	Mis	Delet (0)	Prob_dam (0.999)	0.0002	0.0001603	VUS	-	25.1		Partial	3
9	*CEP152*	NM_001194998.2:c.1499A>T	NP_001181927.1:p.Glu500Val	Mat	Comp het	Mis	Delet (0.01)	Ben (0.159)	0.0002	-	-	-	23.2		Partial	3
10	*CEP290*	NM_025114.4:c.5932C>T	NP_079390.3:p.Arg1978Ter	Bi-parental	Hom	SG	-	-	-	0.00001212	Path	26092869	42		Full	5
11	*MYCN*	NM_005378.6:c.1006del	NP_005369.2:p.Ser336LeufsTer15	De Novo	Het	FS	-	-	-	-	-	-	-		Full	5
12	*ARMC9*	NM_001271466.4:c.879G>A	NP_001258395.2:p.Thr293=	Bi-parental	Hom	Syn	-	-	-	0.000008065	Path, Lik_Path	29159890, 17576681, 9536098	26.8	DS_DL 0.9	Full	5
13	*TUBA1A*	NM_006009.4:c.641G>T	NP_006000.2:p.Arg214Leu	De Novo	Het	Mis	Delet_low conf (0.03)	Ben (0.023)	-	-	Path, Lik_Path	25741868	23.5		Poor	5
14	*WDR19*	NM_025132.4:c.1630_1639del	NP_079408.3:p.Val544LeufsTer72	Mat	Comp het	Spl A	-	-	-	-	-	-	-		Full	5
14	*WDR19*	NM_025132.4:c.817A>G	NP_079408.3:p.Asn273Asp	Pat	Comp het	Mis	Tol (0.28)	Ben (0.003)	-	0.00001629	VUS, Path	29068549	14.46		Full	5
15	*RHO*	NM_000539.3:c.133T>C	NP_000530.1:p.Phe45Leu	Unk	Het	Mis	Tol (0.53)	Prob_dam (0.979)	0.0002	0.00002386	Path	1862076	23.6		Full	5
16	*STAG1*	NM_005862.3:c.1033G>A	NP_005853.2:p.Glu345Lys	De Novo	Het	Mis	Delet (0)	Prob_dam (0.954)	-	-	-	-	28.6		Full	3
17	*BBS1*	NM_024649.5:c.1169T>G	NP_078925.3:p.Met390Arg	Bi-parental	Hom	Mis	Delet (0)	Ben (0.347)	0.001	0.001512	Path, Lik_Path	15 PMIDs	26.2		Full	5
18	*RERE*	NM_001042681.2:c.4286A>T	NP_001036146.1:p.His1429Leu	De Novo	Het	Mis	Delet_low conf (0)	Prob_dam (0.936)	-	-	-	-	28		Full	4
19	*ALMS1*	NM_001378454.1:c.4681_4687dup	NP_001365383.1:p.Ile1563AsnfsTer20	Bi-parental	Hom	FS	-	-	-	-	-		-	-	Full	5
20	*BBS2*	NM_031885.5:c.471+1G>C		Unk	Comp het	Spl d	-	-	-	-	-	-	33	DS_DL 0.95	Full	4
20	*BBS2*	NM_031885.5:c.646C>T	NP_114091.4:p.Arg216Ter	Unk	Comp het	SG	-	-	-	0.00001194	Path, Lik_Path	11567139	37		Full	4

21	*KAT6A*	NM_006766.5:c.1121C>T	NP_006757.2:p.Ser374Leu	Unk	Het	Mis	Delet (0)	Prob_dam (0.966)	-	0.00006369	-	-	28.4		Partial	3
21	*LAMA1*	NM_005559.4:c.3397C>T	NP_005550.2:p.Arg1133Ter	Unk	Comp het	SG	-	-	-	0.00001997	Path	-	38		Full	4
21	*LAMA1*	NM_005559.4:c.281A>G	NP_005550.2:p.Gln94Arg	Unk	Comp het	Mis	Delet (0)	Prob_dam (1)	-	-	-	-	26.4		Full	3
22	*MKKS*	NM_170784.2:c.1017_1018del	NP_740754.1:p.Ile339MetfsTer3	Bi-parental	Hom	FS	-	-	-	-	-	-	-		Full	5
23	*CEP290*	NM_025114.4:c.5668G>T	NP_079390.3:p.Gly1890Ter	Pat	Comp het	SG	-	-	0.0002	0.00009486	Path	18414213, 26092869, 16682970, 16682973, 17564967	36		Full	5
23	*CEP290*	NM_025114.4:c.104T>G	NP_079390.3:p.Val35Gly	Mat	Comp het	Mis	Delet (0)	Prob_dam (0.943)	-	-	-	-	33		Full	3
25	*RAI1*	NM_030665.4:c.2479C>T	NP_109590.3:p.Gln827Ter	De Novo	Het	SG	-	-	-	-	-	-	35		Full	5
26	*PROM1*	NM_006017.3:c.1354dup	NP_006008.1:p.Tyr452LeufsTer13	Unk	Hom	FS	-	-	-	0.0002195	Path, Lik_Path	-	32		Full	5
27	*SETD2*	NM_014159.7:c.5218C>T	NP_054878.5:p.Arg1740Trp	1 parent's unk, absent from other	Het	Mis	Delet (0)	Prob_dam (0.998)	-	-	Path, Lik_Path, VUS	-	32		Full	5
28	*OPA1*	NM_130837.3:c.2678del	NP_570850.2:p.His893LeufsTer9	1 parent's unk, absent from other	Het	FS	-	-	-	-	-	-	-		Partial	5
29	*ALMS1*	NM_015120.4:c.11881dup	NP_055935.4:p.Ser3961PhefsTer12	Unk	Comp het	FS	-	-	-	-	-	-	-		Full	5
29	*ALMS1*	NM_015120.4:c.1241-81_1252delinsCCTGCAGGCCCTCCACATATGCTACAAAATA	-	Unk	Comp het	Spl A	-	-	-	-	-	-	-		Full	4
30	*PHIP*	NM_017934.7:c.3202delAinsTACCTG	NP_060404.4:p.Ile1068AsnfsTer3	Unk	Het	FS	-	-	-	-	-	-	-		Full	5
31	*RAB28*	NM_001017979.3:c.58dup	NP_001017979.1:p.Asp20GlyfsTer62	Unk	Hom	FS	-	-	-	0.000004504	-	-	33		Full	5
32	*ALMS1*	GMC questionnaire reports		Unk	Comp het	Exon del	-	-	-	-	-	-	-		False positive	False positive

		exon 11 Deletion														
32	*ALMS1*	NM_015120.4:c.9001C>T	NP_055935.4:p.Gln3001Ter	Unk	Comp het	SG	-	-	-	0.000004008	-	-	36		Full	5
34	*POLA1*	NM_001330360.2:c.460G>T	NP_001317289.1:p.Asp154Tyr	Mat	XLR	Mis, male	Delet (0)	Prob_dam (0.935)	-	0.0001049	-	-	32		Partial	3
41	*CSPP1*	NM_001382391.1:c.2968+5G>A		Mat	Comp het	Spl region	-	-	-	-	VUS	-	-	DS_DL 0.79	Full	3
41	*CSPP1*	GrCh38: Chr8:67136672_67139048del		Pat	Comp het	2.7kb del incl exon 15									Full	5
42	*PIBF1*	NM_006346.4:c.1205T>C	NP_006337.2:p.Met402Thr	Unk	Comp het	Mis	Tol (0.09)	Ben (0.157)	-	-	-	-	22.8		Full	3
42	*PIBF1*	GrCh38: chr13:g.72783352_72796671del		Unk	Comp het	13.3kb del incl exons 2-4									Full	5
48	*LRRC45*	NM_144999.4:c.1074_1075insTG	NP_659436.1:p.Leu359CysfsTer19	Bi-parental	Hom	FS	-	-	-	-	-	-	-		Candidate gene	Candidate gene
56	*BBS9*	NM_198428.3:c.1028G>A	NP_940820.1:p.Gly343Glu	1 parent's unk, present in other	Hom	Mis	Delet (0)	Prob_dam (1)	-	-	-	-	25.9		Full	3
61	*WT1*	NM_024426.6:c.1400G>A	NP_077744.4:p.Arg467Gln	Unk	Het	Mis	Delet_low conf (0.02)	Poss_dam (0.767)	-	-	Path	1302008	29.9		Full	5
67	*ALMS1*	NM_015120.4:c.1612C>G	NP_055935.4:p.Leu538Val	Pat	Comp het	Mis	Tol_low conf (0.17)	Ben (0.009)	-	0.0003289	lik_ben, VUS	-	0.003		Full	3
67	*ALMS1*	NM_015120.4:c.9613A>G	NP_055935.4:p.Ile3205Val	Mat	Comp het	Mis	Delet (0.03)	Prob_dam (0.997)	-	0.000008021	VUS	-	23		Full	3
69	*BBS1*	NM_024649.5:c.1169T>G	NP_078925.3:p.Met390Arg	Mat	Comp het	Mis	Delet (0)	Ben (0.347)	0.001	0.001512	Path, Lik_Path	15 PMIDs	26.2		Full	5
69	*BBS1*	NM_024649.5:c.592-1333_831-449del		Pat	Comp het	4.7kb del incl exons 8 and 9									Full	5
71	*BCORL1*	NM_001379451.1:c.3463C>A	NP_001366380.1:p.Pro1155Thr	Mat	XLR	Mis, male	Tol (0.12)	Poss_dam (0.557)	-	0.00003468	-	-	21.2		Partial	3
72	*CHD4*	NM_001273.5:c.3225G>T	NP_001264.2:p.Met1075Ile	1 parent's unk, absent from other	Het	Mis	Delet (0)	Ben (0.358)	-	-	-	-	26		Full	3
75	*BBS4*	NM_033028.5:c.642+3A>T		1 parent's unk, present in other	Hom	Spl region	-	-	-	-	-	-	22.5	DS_DL 0.82	Data missing	3

76	*BBS10*	NM_024685.4:c.1790G>A	NP_078961.3:p.Gly597Asp	1 parent's unk, present in other	Hom	Mis	Delet (0)	Prob_dam (0.999)	-	-	-	-	25.8		Full	3
82	*IDUA*	NM_000203.5:c.1469T>C	NP_000194.2:p.Leu490Pro	Unk	Comp het	Mis	Delet (0.02)	Ben (0.086)	-	0.00004681	Path	23757202, 7550232	18.91		Full	5
82	*IDUA*	NM_000203.5:c.406G>A	NP_000194.2:p.Ala136Thr	Unk	Comp het	Mis	Delet (0.01)	Ben (0.085)	-	0.0001039	VUS	-	20.7		Full	3

Variants are described using Human Genome Variation Society (HGVS) nomenclature with the RefSeq transcript selected (or longest transcript if RefSeq not available). They are accompanied by the GEL tier, zygosity (presumed where segregation data unavailable), mutational mechanism, ACMG classification, match to phenotype and assigned diagnostic confidence.

Abbreviations: mat = maternal, pat = paternal, unk = unknown, AF = allele frequency, het = heterozygous, comp het = compound heterozygous, XLR = X-linked recessive, XLD = X-linked dominant, hom = homozygous, spl = splice, Spl D = splice donor, Spl A = splice acceptor, Syn = synonymous, SG = stop gain, FS = frameshift, del = deletion, kb = kilobase, low_conf = low confidence, Tol = Tolerated, delet = deleterious, Prob_dam = probably damaging, ben = benign, poss_dam = possibly damaging, incl = including, Path = Pathogenic, Lik_Path = likely Pathogenic, VUS = variant of uncertain significance, lik_ben = likely benign, PMID = PubMed identifier, CADD = Combined Annotation Dependent Depletion (CADD), DS = delta score, DS_DL = delta score donor loss,

ACMG classifications: Class 5 = pathogenic, Class 4 = likely pathogenic, Class 3 = VUS

References

1. Gee HY, Otto EA, Hurd TW, Ashraf S, Chaki M, Cluckey A, et al. Whole-exome resequencing distinguishes cystic kidney diseases from phenocopies in renal ciliopathies. Kidney Int. 2014;85(4):880-7.
2. Knopp C, Rudnik-Schoneborn S, Eggermann T, Bergmann C, Begemann M, Schoner K, et al. Syndromic ciliopathies: From single gene to multi gene analysis by SNP arrays and next generation sequencing. Molecular and cellular probes. 2015;29(5):299-307.
3. Bachmann-Gagescu R, Dempsey JC, Phelps IG, O'Roak BJ, Knutzen DM, Rue TC, et al. Joubert syndrome: a model for untangling recessive disorders with extreme genetic heterogeneity. Journal of medical genetics. 2015;52(8):514-22.
4. Sawyer SL, Hartley T, Dyment DA, Beaulieu CL, Schwartzentruber J, Smith A, et al. Utility of whole-exome sequencing for those near the end of the diagnostic odyssey: time to address gaps in care. Clinical genetics. 2016;89(3):275-84.
5. Braun DA, Schueler M, Halbritter J, Gee HY, Porath JD, Lawson JA, et al. Whole exome sequencing identifies causative mutations in the majority of consanguineous or familial cases with childhood-onset increased renal echogenicity. Kidney Int. 2016;89(2):468-75.
6. Kang HG, Lee HK, Ahn YH, Joung JG, Nam J, Kim NK, et al. Targeted exome sequencing resolves allelic and the genetic heterogeneity in the genetic diagnosis of nephronophthisis-related Ciliopathy. Exp Mol Med. 2016;48(8):e251.
7. Watson CM, Crinnion LA, Berry IR, Harrison SM, Lascelles C, Antanaviciute A, et al. Enhanced diagnostic yield in Meckel-Gruber and Joubert syndrome through exome sequencing supplemented with split-read mapping. BMC Medical Genetics. 2016;17(1):1.
8. Castro-Sánchez S, Álvarez-Satta M, Tohamy MA, Beltran S, Derdak S, Valverde D. Whole exome sequencing as a diagnostic tool for patients with Ciliopathy-like phenotypes. PLoS One. 2017;12(8):e0183081.
9. Vilboux T, Doherty DA, Glass IA, Parisi MA, Phelps IG, Cullinane AR, et al. Molecular genetic findings and clinical correlations in 100 patients with Joubert syndrome and related disorders prospectively evaluated at a single center. Genet Med. 2017;19(8):875-82.
10. Shamseldin HE, Al Mogarri I, Alqwaiee MM, Alharbi AS, Baqais K, AlSaadi M, et al. An exome-first approach to aid in the diagnosis of primary ciliary dyskinesia. Hum Genet. 2020;139(10):1273-83.
11. Shamseldin HE, Shaheen R, Ewida N, Bubshait DK, Alkuraya H, Almardawi E, et al. The morbid genome of ciliopathies: an update. Genet Med. 2020.
12. Hammarsjö A, Pettersson M, Chitayat D, Handa A, Anderlid BM, Bartocci M, et al. High diagnostic yield in skeletal ciliopathies using massively parallel genome sequencing, structural variant screening and RNA analyses. J Hum Genet. 2021.
13. Forsythe E, Beales PL. Bardet-Biedl syndrome. European journal of human genetics : EJHG. 2013;21(1):8-13.
14. Bachmann-Gagescu R, Dempsey JC, Bulgheroni S, Chen ML, D'Arrigo S, Glass IA, et al. Healthcare recommendations for Joubert syndrome. Am J Med Genet A. 2020;182(1):229-49.
15. Hartill V, Szymanska K, Sharif SM, Wheway G, Johnson CA. Meckel-Gruber Syndrome: An Update on Diagnosis, Clinical Management, and Research Advances. Front Pediatr. 2017;5(244).
16. Quinlan RJ, Tobin JL, Beales PL. Modeling ciliopathies: Primary cilia in development and disease. Curr Top Dev Biol. 2008;84:249-310.
